# Reconfigurable Vortex-like Paramagnetic Nanoparticle Swarm with Upstream Motility and High Body-length Ratio Velocity

**DOI:** 10.34133/research.0088

**Published:** 2023-03-27

**Authors:** Luyao Wang, Han Gao, Hongyan Sun, Yiming Ji, Li Song, Lina Jia, Chutian Wang, Chan Li, Deyuan Zhang, Ye Xu, Huawei Chen, Lin Feng

**Affiliations:** ^1^ School of Mechanical Engineering and Automation, Beihang University, Beijing 100191, China.; ^2^ Beijing Advanced Innovation Center for Biomedical Engineering, Beihang University, Beijing 100191, China.; ^3^ Center of Soft Matter Physics and Its Applications, Beihang University, Beijing 100191, China.

## Abstract

Drug delivery systems with high-targeted doses can minimize excipients, reduce side effects, and improve efficacy. Human blood circulation is a complex circulatory system, and the motion control of microrobots in the static flow field in vitro is completely different from in vivo. How to achieve precise counterflow motion for targeted drug delivery without vascular blockage and immune rejection is the biggest challenge for micro-nano robots. Here, we propose a control method that enables vortex-like paramagnetic nanoparticle swarm (VPNS) to move upstream against the flow. By mimicking the clustering motion of wild herring schools and the rolling of leukocytes, VPNS are incredibly stable even when subjected to high-intensity jet impacts in the blood environment, can travel upstream, anchor at the target location, and dissipate when the magnetic field is withdrawn, which greatly reduces the risk of thrombosis. VPNS can also upstream along the vessel wall without an additional energy source and has a marked targeted therapeutic effect on subcutaneous tumors.

## Introduction

Untethered micro-nano robots are expected to be the next generation of minimally invasive therapeutic instruments in biomedicine, serving as powerful medical vehicles that can access hard-to-reach areas of the body to perform least-invasive procedures and drug delivery [1–3]. The motions of microrobots in liquid media are at a low Reynolds number condition, and according to the scallop theorem [4,5], movement in such a super-viscous environment requires the breaking of the time-reversal symmetry of the kinetic patterns. Many noncontact microrobot-driven modalities, such as photoelectric driving [6], ultrasounds [7], magnetic fields [8], and their combinations [9], have been reported. For drug delivery, the circulatory system is the ideal pathway for navigation and deployment to all organs and tissues at depth [10]; however, large blood cells, viscous proteins, and muscular jet impingement in the vasculature severely hinder active movement and work execution of microrobots. Existing microrobot applications in vivo are primarily limited to superficial tissues (e.g., eyes [11,12]), subcutaneous tumors [13], and digestive system (e.g., gastrointestinal tract [14]), which are relatively easy to access, observe, and control, owing to the absence of flow fluid perturbations. However, the blood circulation system is inescapable when microrobots are required in deeper organs and tissues.

Under natural conditions, leukocytes are the only cells that can autonomously move in a crowded and heterogeneous bloodstream [15], which is facilitated by the presence of a cell-free layer on the vessel wall [16], the attenuation of fluid velocity, and the decline in crowding due to vessel wall marginalization [17], and wall-adherent rolling, which exhibits superior performance to swimming in the center of a vessel [18]. However, deactivated microrobots in a Poiseuille flow are subjected to lift forces caused by an imbalance in the fluid shear distribution, which migrate the microrobots toward the center of the tunnel [19]. In the current artificial system, the counterintuitive near-wall upstream migration of the microrobots is mainly restricted to chemically catalyzed motors and additional auxiliary energetic outfields such as ultrasound, optical and chemical propulsion, etc. Katuri et al. [20] demonstrated that Janus particles catalyzed by hydrogen peroxide exhibit cross-flow migration. Nelson et al. [21,22] directed the migration of microrobots to the vessel wall for upstream movement using ultrasonic nodes. Sitti et al. [23,24] developed a Janus microparticle that relies on gravitational sinking and external magnetic field navigation to reverse flowing blood migration. These various delicate individual microrobots, primarily fabricated using micro-electromechanical systems and chemistry, have made numerous important advances in realizing low-Reynolds-number fluid environments. These microrobot swarms with colony-intelligent behavior are more flexible and versatile in shape, and they can satisfy the demands of multiple complex tasks, such as navigating uneven surfaces, complex mazes, highly elevated steps, and wide trenches [25]. The deformation and motility are beyond the capabilities of a single rigid microrobot. Notably, unlike sophisticated macroscopic robots capable of carrying onboard power and sensing components, external field-driven micro-nanorobots can only respond indiscriminately to field modifications, which presents a challenge for the proposed clustering strategy. Additionally, the upstream clustering control of magnetic nanoparticles (MNPs) with biocompatibility with physiological fluids requires further research, owing to the potential for blockage and rejection in the vasculature caused by large microrobots.

**Fig. 1.**
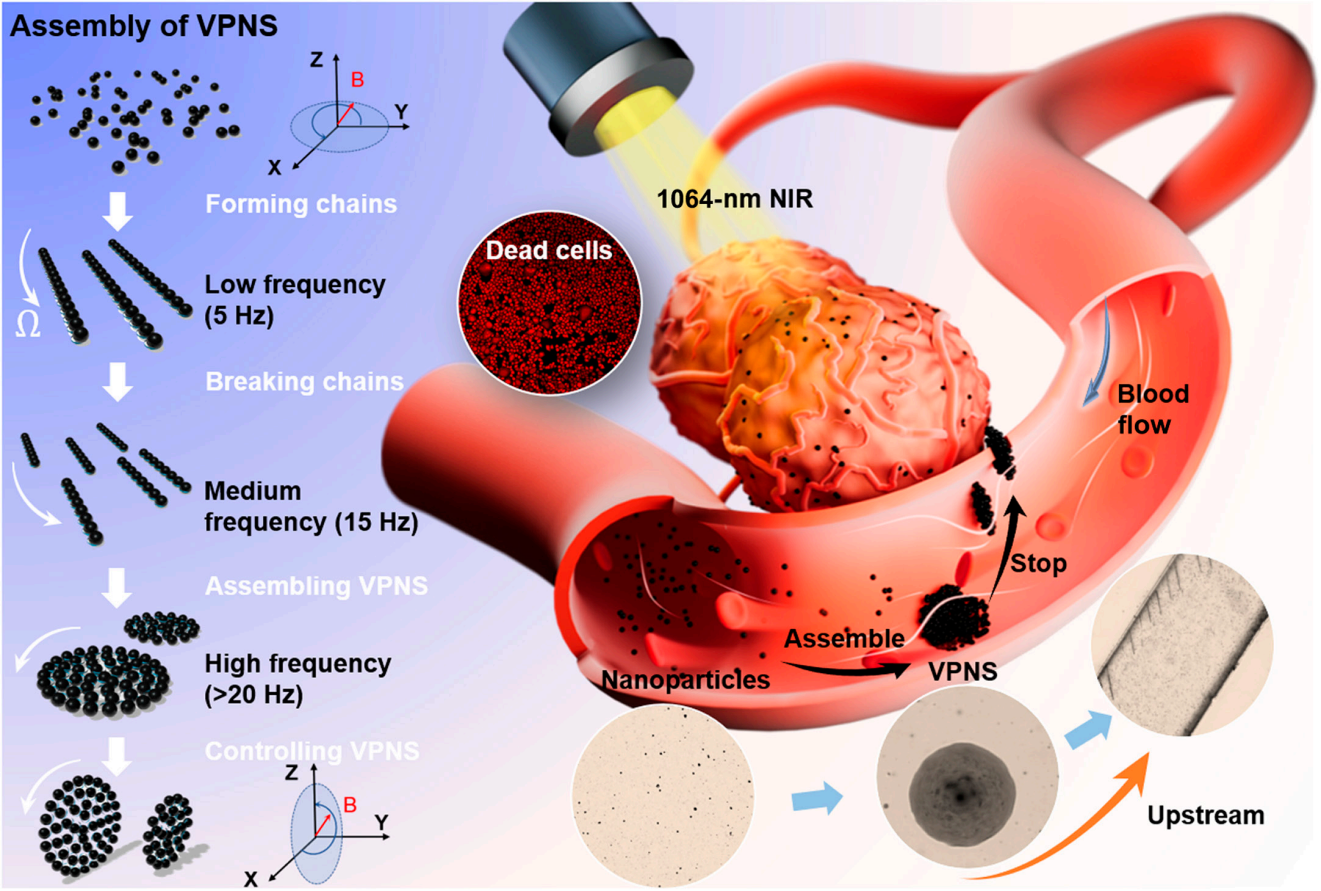
Conceptual diagram of VPNS collection and upstream motion.

Herein, we report a vortex-like paramagnetic nanoparticle swarm (VPNS) dynamically assembled from MNPs with a diameter of 300 nm as the minimum unit (the material used is Fe_3_O_4_). Inspired by the clustered movement patterns of wild herring schools and the appositional migration of leukocytes in the bloodstream, an innovative 3-dimensional (3D) tilted rotating magnetic field is applied. MNPs can transform from a loose suspension state into a swarm that combines liquid deformability and solid robustness. The integrated VPNS can maintain the stability of their vortex swarm structure under high-intensity jet impingements in crowded distinctive environments. Further, they exhibit kinematic properties in which they tend to migrate upstream toward the vessel wall, as shown in Fig. 1. Wall tending is dependent on a sharp increase in the linear velocity at the edge of the swarm, which is induced by a high-frequency magnetic field (>100 Hz). When the linear velocity exceeds the fluid shear flow velocity, the resulting Saffman lift drags the VPNS to the wall [26], which achieves simultaneous VPNS toward the wall and upstream motion relying only on the energy input from the magnetic field. The assembly behavior of a VPNS is flexibly reconfigurable; thus, a VPNS can be immediately dissolved into monodisperse particles and then reconstituted into other morphological forms as required. Compared with previous studies, our research on VPNS is equipped with a dual closed-loop control module based on visual recognition, which can control the position error of in vitro experiments to within 20 μm, and the high-precision in vitro control capability is expected to provide a solid foundation for future targeted therapies. In addition, the coupling of multiple functions such as high-speed upstream, target anchoring, and dissolution reconstruction can be achieved simply by programming the electromagnetic field alone, which has important practical value for simplifying control systems and improving control efficiency. Movement in the vasculature with a stable swarm pattern can enhance the contrast of various biomedical imaging, allowing easy identification and navigation, such as ultrasound imaging, magentic resonance imaging, magnetic particle imaging, etc. Aggregating all the nanoparticles required for therapy into a swarm enhances their ability to deliver drugs, perform tasks, and adapt to complex environments, especially in blood with flowing blood cells rushing around, and the free and controlled movement of nanoparticles benefits from the clustering mode. Driven by a biologically safe magnetic field with an intensity of approximately 6 mT, the VPNS possesses a relative movement speed above 100 μm/s, approximately 340 times its smallest constituent unit; higher velocities may be achieved by increasing the frequency and intensity of the magnetic field. Finally, the photothermal effect of MNPs is exploited to induce apoptosis in mouse breast carcinoma cells (4T1 cells), and VPNS also showed highly targeted and ablative effects on mouse tumors in in vivo experiments. The successful validation of the proposed VPNS upstream and reconfiguration strategy represents a step forward in utilizing micro-nanorobots in blood flow applications.

## Results

### Formation and motion control of VPNS

**Fig. 2.**
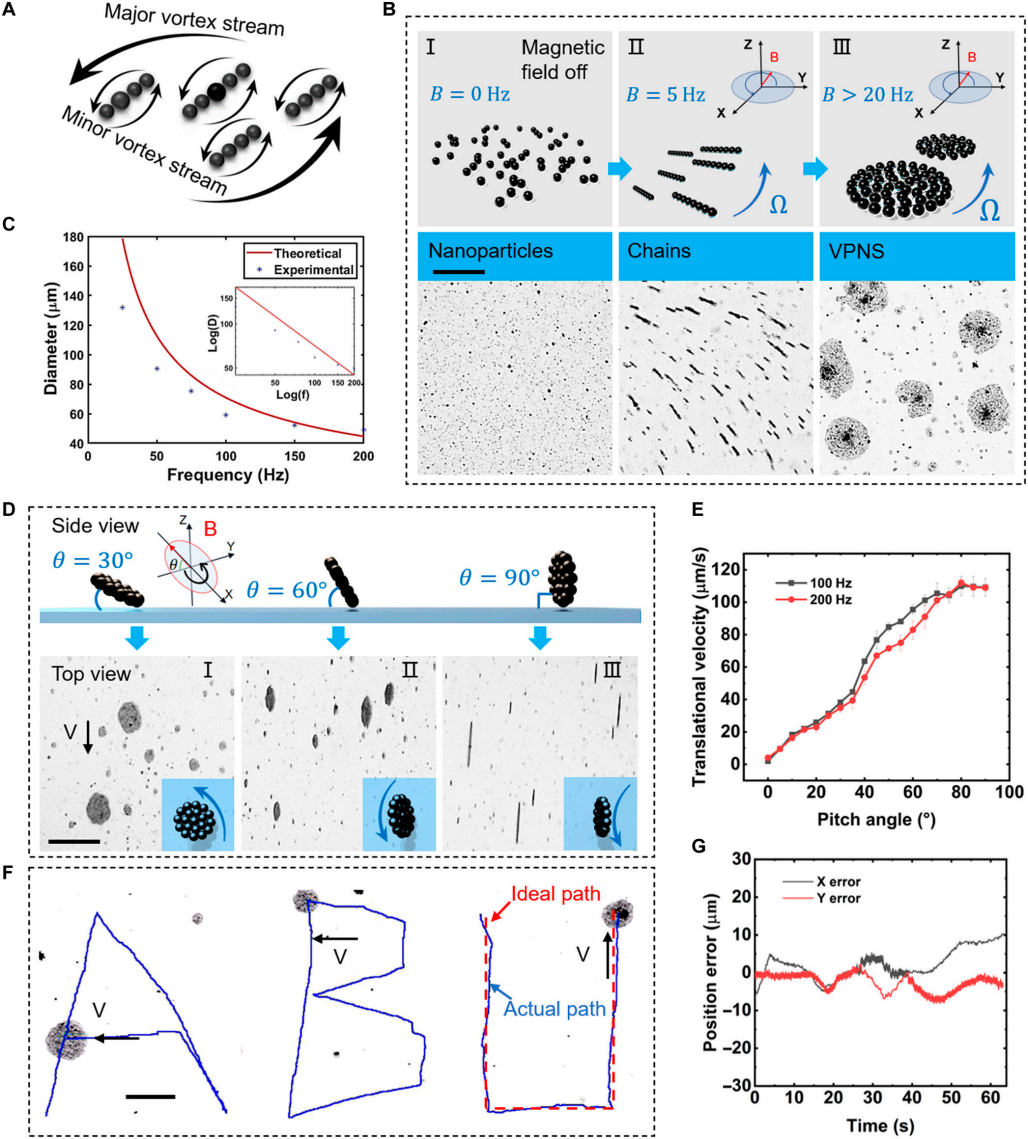
The formation process and precise motion control of VPNS. (A) Schematic of the incorporation process of MNPs induced by a rotating chain of paramagnetic nanoparticles. The larger diameter particles in the middle that function as corotating centroids, and the thicker arrows denote flows with higher velocities. (B) The formation process of VPNS: (I) MNPs with diameters of 1,000 and 300 nm were dispersed in the PBS solution. (II) Stabilized short chains developed when the rotating magnetic field frequency reaches 5 Hz. (III) Beyond a frequency of 20 Hz, short chains polymerize into VPNS. The scale bar is 20 μm. (C) Comparison of theoretical and experimental stable cluster size in a plane. (D) VPNS projection morphology and velocity vary with the pitch angle of the magnetic field (𝜃): (I) Pith angle θ = 30^°^; (II) θ = 60^°^; (III) θ = 90^°^. The scale bar is 20 μm. (E) Statistics on the relationship between the translational velocity of VPNS and the pitch angle of the rotating magnetic field. (F) Precise path tracking for VPNS: letters ABU. The red dashed line indicates the ideal path in the U-shaped path, and the blue line indicates the actual motion path. The scale bar is 50 μm. (G) Position error of U-shaped path feedback control.

The generation of VPNS depends on the local vortex that is excited by a rotating short particle chain [27]. In this study, 2 differently sized magnetic particles of Fe_3_O_4_ were used to demonstrate and control the formation of VPNS. Nanoparticles with superparamagnetic properties exhibit a homogeneous and loose liquid-like state in the absence of an externalmagnetic field, and they are persistently subject to irregular motion in the local area surrounding them owing to Brownian forces (Fig. 2B [I]). The paramagnetic nanoparticles are magnetized by applying a driving magnetic field, and each magnetic particle is considered a magnetic dipole. The dipole-dipole force mutually attracts the MNPs, and they tend to be arranged in chains along the magnetic field direction. The short chains that are formed under dipole forces rotate along with the magnetic field and trigger local vortices around the rotating short chains, as shown in Fig. S7. The sharpest fluid velocities and pressure differences are produced adjacent to the 2 edges of the short chain (or short-chain cluster), whereas the central region of the short chain has almost negligible fluid velocities. The color-gradient legend in the figure indicates the magnitude of the streaming velocity, and the black lines and arrows represent the direction of the flow, that is, the vortex. Under the action of the rotating magnetic field excited in 3D space, the MNPs at a larger scale can produce more powerful vortices, making it possible to exert a mutual attraction force on other particles and chains at a further distance. Therefore, with the powerful vortex induced by large-diameter particles, the chains decrease their distance and approach each other while rotating around their center; when the separation of the chains is narrower than the critical value, the merging of the vortex occurs, and the chain starts to perform the coaxial motion, as shown in Fig. 2A.

The stability of short chains depends primarily on the magnetic field strength and rotation frequency. The Mason number [21,28] is typically employed to characterize the stability of magnetic particle chains:

$$M_{n}=\frac{\mu \omega }{\mu_{0}\chi_{p}H^{2}}\frac{n^{3}}{\left(n-1\right)\left[\text{ln}\left(n/2\right)+2.4/n\right]},$$ (1)

which is defined as a dimensionless parameter that expresses the ratio of the magnetic and viscous forces of the MNPs. At a low *Mn* (set the magnetic field frequency to 5 Hz), the magnetic force acting on the MNPs exceeds the viscous force, and the particles aggregate into chains (Fig. 2B[II]); however, as *Mn* increases (i.e., the magnetic field rotation frequency), the mechanical forces applied to the MNPs tend to decrease to form chains of a smaller length, as shown in Fig. S8. When preserving the magnetic field strength constant, a conscious change in the magnetic field frequency can manipulate the MNP swarms toward the desired morphology. The continuous presence of a high-frequency rotating magnetic field (beyond 20 Hz, details in Note S1) induces a constant merging of the vortices between the chains, and the interfused vortices can generate a more energetic kinetic system. An inadequate distance between the chains drives them to impinge on each other. The entire short chains are disrupted but still bound in the hydrodynamic system of the central vortex, which is composed of 1,000-nm-diameter particles as vortex nuclei. The remaining 300-nm-diameter particles are compactly confined in the surrounding area, and the transformation process from short chains to vortices is completed almost instantaneously. The transformation control is shown in Fig. 2B(III), where the black particles at the core of the VPNS are MNPs with a diameter of 1,000 nm, and the surrounding pale-colored surrounding objects are composed of MNPs with a diameter of 300 nm. The abovementioned VPNS is formulated under a rotating magnetic field:

$$B_{\left(t\right)}=B_{x}\text{cos}\left(2\pi \text{ft}\right)e_{x}-B_{y}\text{sin}\left(2\pi \text{ft}\right)e_{y},$$ (2)

where ***e****_x_*, ***e****_y_* denote the unit vectors along the X- and Y-axis in the Cartesian coordinate system, respectively; f is the rotational frequency of the magnetic field; and *B_x_*, *B_y_* represent the intensity amplitude of the input magnetic field. To execute the controllable locomotion of the VPNS, the magnetic field rotating in the XY plane must be “erected” to produce a component in the Z-direction. Here, we define the tilt angle of the rotating magnetic field as follows:

$$\theta ={\text{tan}}^{-1}\sqrt{B_{x}^{2}+B_{y}^{2}}/B_{z},$$ (3)

which represents the angle of the rotating magnetic field plane for the XY plane, the angle between the rotating magnetic field intensity vector *B* and the Z-axis. The component of the magnetic field intensity vector *B* along the Z-axis is used to produce the dominant vortex to maintain the stability of the vortex swarms, and the component in the XY plane controls the motion orientation of the vortex swarm robots. Both of these components coordinate mutually to govern the stabilization and motion speed of the VPNS. When the particle cluster is regarded as a rigid disk, the particle at the edge conforms to the law of circular motion, and the component of the magnetic force along the central line provides the centripetal force. Assuming that the direction of the magnetic force between particles 1 and 2 is along the centerline, the cluster size can be obtained as

$$R=\sqrt[{3}]{\frac{9\mu_{0}m_{2}^{2}}{8{\pi \rho }_{p}a^{5}\Omega^{2}}},$$ (4)

where *m*_2_, *ρ_p_*, *a* represent the magnetic moment, density, and radius of the particle, respectively; *Ω* is magnetic field angular velocity; and *μ*_0_ is the vacuum permeability. The comparison of the theoretical results and experimental data in Fig. 2C indicates that frequency increases follow the overall trend while the cluster size decreases correspondingly, confirming that the theoretical model is valid. However, the theoretical and experimental values significantly differ in low frequencies. A possible reason is that the low surface fraction of particles leads to an unsaturated cluster (details in Note S2).

By changing the pitch angle of the magnetic field, the posture and direction of the VPNS can be precisely adjusted. Figure 2D illustrates the positional transformation of the microrobot under different input magnetic field pitch-angle (*θ*) conditions. The VPNS gradually deforms from a regular circle to an elliptical state as the pitch angle increases. This occurs because the vortex will be inclined with the magnetic field as an integrative motion system. When the rotating magnetic field gradually transforms from XY in-plane rotation to 3D space tilt rotation, the VPNS is gradually “erected” from its initial flat state until the pitch angle reaches the maximum value (90°). Then, the VPNS is entirely upright and perpendicular to the XY plane, which looks like a thin rotating “tire” from the side. The increase in the pitch angle indicates that the rotating magnetic field vector is distributed to more components in the XY plane, which causes the magnetic field strength to regulate the velocity of the VPNS to increase. As shown in Fig. 2E, the translational velocity of the VPNS increases with the pitch angle of the input magnetic field and eventually smooths out. The magnetic field strength applied when implementing the path-tracking task of the VPNS is approximately 6 mT (the control system is shown in Fig. S16); by optimizing the pitch and yaw angles of the magnetic field, we can precisely guide the VPNS to travel along a preset path. As shown in Fig. 2F, the VPNS completes the tracking of the sophisticated path “ABU” when the input rotating magnetic field pitch angle *θ* is 10°, and the frequency is 100 Hz. Using the self-developed vision feedback module, it is possible to control the linear motion position error of the VPNS below 10 μm (path “U”), which is only approximately one-third of its diameter (Fig. 2G). This demonstrates the superior controlled micromotion capability of the solenoid system and microrobot. By considering the VPNS developed in the low-Reynolds-number regime as a rotating disk, the positional posture of the VPNS is consistent with the rotating magnetic field.

### Reconfigurable upstream motility and dynamic analysis of VPNS

To investigate the upstream motility and reconfigurability of the VPNS, we conducted controlled experiments in a microfluidic chip with a square cross-section (300 × 300 μm), and a suspension of MNPs at a concentration of 0.5 mg/ml was pumped through at a volume flow rate of 1 μl/min. When the rotating magnetic field was applied with a pitch angle of 90° (i.e., the VPNS “rolling” near the wall of the microfluidic channel as a reconfigurable integral entity), the presence of the wall broke the kinematic symmetry of the VPNS in a low-Reynolds-number fluid environment [29]. We assume that the area of the fluid–wall contact is a lubrication boundary layer that belongs to the region experienced by higher shear in the fluid [30]. The imbalance between the shear at the top and bottom of the VPNS (hydrodynamic mobility mismatch) generates pure propulsion, which contributes to converting the rotational motion of the microrobotic system into translational play (Fig. S17). From Fig. 2E, it can be concluded that at magnetic field frequencies of 100 and 200 Hz, the translational velocity of the VPNS increases with the pitch angle of the magnetic field and reaches a peak at a pitch angle of 90°, at which the VPNS is entirely upright and becomes a rolling “disc”, which appears spindle-shaped when visualized from an overhead perspective (as shown in Fig. S18). The morphology and posture of the VPNS contributed critically to reducing the viscous drag of the movement in a hydrodynamic environment (Fig. S19).

**Fig. 3.**
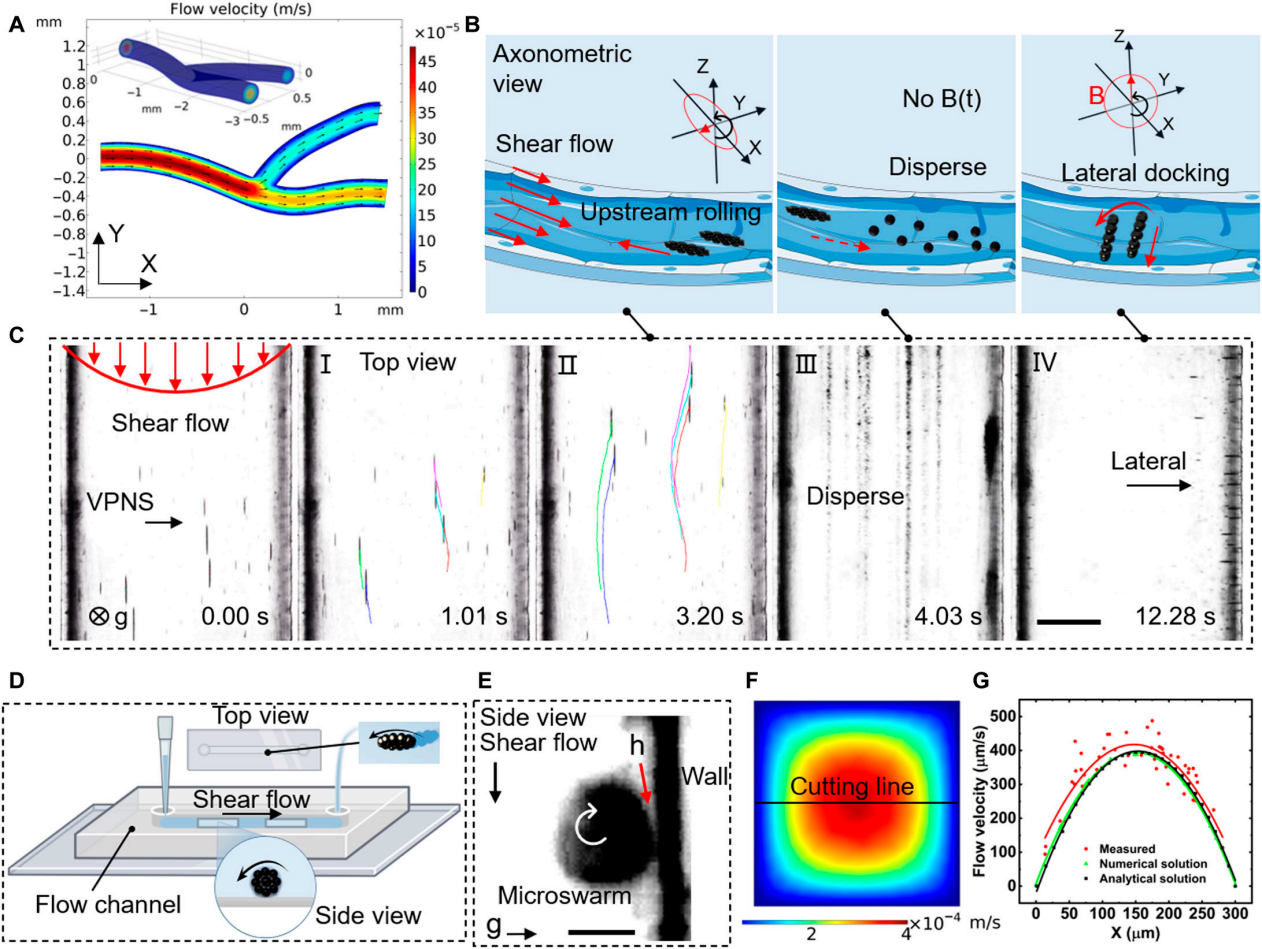
Reconfigurable upstream motility of VPNS. (A) Simulation of blood flow in a 3D vessel. (B) Schematic diagram of the formation and upstream motion of VPNS in the flow field. (C) Top view: VPNS population against Poiseuille flow in a microchannel, where the color curves represent the VPNS motion path traced using ImageJ. (I and II) Upstream motion parallel to the direction of hydrodynamic flow. (III) Dissolution of VPNS after withdrawal of the magnetic field. (IV) Nanoparticles recombine into VPNS via lateral movement and hovering toward the side of the microfluidic channel. The scale bar is 100 μm. (D) The top and side views of VPNS in the microfluidic channel. (E) Side view of VPNS; h is the gap between the VPNS and the wall. Scale bar is 30 μm. (F) The fluid velocity distribution cloud over the cross-section of the square flow channel used in the experiment. (G) Fluid velocity distribution curve at the central intersecting line of any cross-section in a microfluidic channel.

Figure 3A is a simulation of the blood flow velocity in the 3D vessel. Figure 3B shows a schematic of the upstream locomotion and reconfiguration of the VPNS. When a rotating magnetic field with a pitch angle of 90° is supplied to the colloidal system, vortex-shaped microrobots begin to form and fall autonomously toward the wall while rotating. They demonstrate upstream motility in the microfluidic chip across a hydrodynamic environment, where the average flow velocity reaches 370 μm/s. It is worth noting that this upstream motility performance is not limited to the low-flow region on either side of the runner, but it is also permitted in the high-flow region in the middle of the chip, as shown by the color curve motion trajectories in Fig. 3C(I) and (II). To cope with the impact and shear of the fluid, the VPNS oscillates laterally as it moves upstream, rather than in the standard straight preset direction. The microrobots roll upstream in a vigorous fluid strike, After approximately 4 s, the VPNS within the visual field travels upwards across the microfluidic chip from one side to the other. At this point, the magnetic field withdraws, and the VPNS instantly dissolves into monodispersed nanoparticles and motion with the fluid flow (Fig. 3C[III]), which means that the VPNS will not cause clogging in the microfluidic channel. The paramagnetic properties of the MNPs suggest that the nanoparticles possess very little remanence and coercivity to maintain the swarm condition without the coercive effect of the magnetic field. Next, we maintained the magnetic field pitch angle at a constant value (90°) and modified the yaw angle to orient the microrobot toward the lateral side of the microfluidic channel (Fig. 3C[IV]). Figure 3D is a schematic diagram of the VPNS movement in the microchannel, including both top and side views. The VPNS that traverses the flow channel laterally is subjected to hydrodynamic shear forces perpendicular to the translational direction and magnetic forces. The fluid velocity and magnetic field precession velocity combine to synthesize the actual motion of the VPNS, i.e., a parabolic-shaped kinematic path, which provides a new inspiration for the kinematic coupling of the microrobot in a flow field environment. An image of the microrobot, captured from the side view (Fig. 3E), confirms the above description of the VPNS morphology (i.e., the rotating propelling spindle-shaped disc).

We use polydimethylsiloxane (PDMS) to fabricate rectangular-cross-section microfluidic chips with an edge length of 300 μm, as shown in Fig. S20. For this particular pipe structure, mathematical models of the flow velocity distribution at any cross-section along the flow direction are obtained by analyzing the fluid in it [31,32], which is as follows:

$$U_{f}(x,y)=\frac{Q}{0.07\mu }[\alpha^{2}-y^{2}+\sum_{n=0}^{\infty }\frac{{\left(-1\right)}^{1+n}\text{cosh}(\pi x^{2})}{2\alpha^{2}{\left(1+2n\right)}^{2}}],$$ (5)

where *Q* is the volumetric flow rate pumped into the microfluidic channel, *μ* is the dynamic viscosity of the water at 25 °C, *α* represents the cross-sectional edge length of the microfluidic channel (details in Note S3), and a standard Cartesian coordinate system is established using the 2 orthogonal edges of the cross-section as XY coordinates. Based on the actual experimental conditions, the cross-sectional flow rate distribution was simulated using COMSOL Multiphysics 5.5 (COMSOL Inc.) for an input volume flow rate of 1 μl/min, as shown in Fig. 3F. The specific analytical solution for the flow velocity distribution is shown in Fig. S21A and B. We choose an intercept line parallel to the X-axis at the center of the section. The data are collected to obtain the velocity distribution of the square tube section as a rotating paraboloid along the centerline, consistent with the Poiseuille flow distribution state in a conventional circular pipe.

**Fig. 4.**
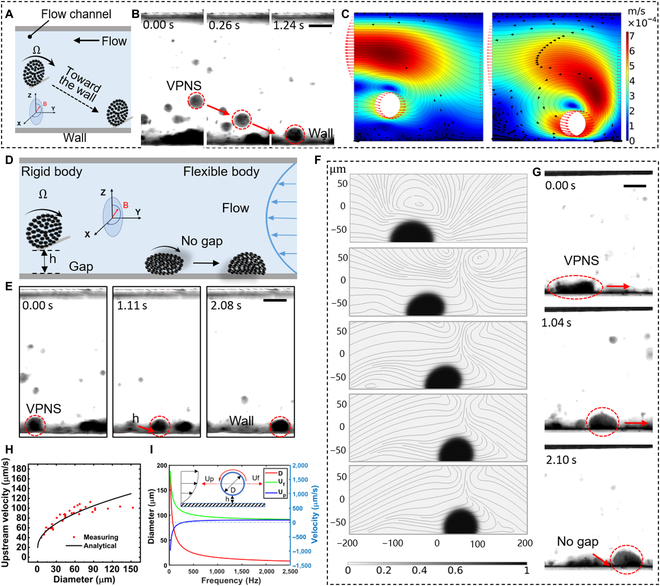
VPNS rotation toward the wall and 2 motion models: rigid and flexible body. (A and B) VPNS rotation toward the wall. The scale bar is 50 μm. (C) Simulation of VPNS rotation toward the wall. (D) Schematic diagram of the rigid- and flexible-body models with upstream motion. (E) Upstream experimental results of the rigid-body model. The scale bar is 50 μm. (F) Upstream simulation results for the flexible-body model. (G) Upstream experimental results of the flexible-body model. The scale bar is 50 μm. (H) The curve of upstream velocity versus diameter for VPNS, where the red dots are the actual measured values, and the black line is the theoretical result. (I) Analysis of theoretical motion parameters of VPNS rigid-body model.

Furthermore, the drifting method was utilized to measure the specific flow velocities in the microfluidic channel and perform data statistics and curve fitting. The discrepancy between the measured flow velocity and the obtained numerical solution was within 6%, as shown in Fig. 3G. With a pumped volume flow rate of 1 μl/min in the flow channel, the maximum flow velocity at the center of the channel can approach 400 μm/s. The self-assembled VPNS has a diameter of approximately 50 μm and is capable of upstream rolling at any location near the vessel wall; in this case, the VPNS is resistant to fluid scour of at least 300 μm/s. Figure 4H shows the upstream velocity of the VPNS versus the swarm diameter. At low flow velocities (i.e., less than 400 μm/s), the upstream rolling is minimally influenced by the externally imposed flow. As the diameter of the VPNS increases, the linear velocity of the cluster edge also increases, which results in an upstream translational velocity growth. However, this growth does not proceed indefinitely, owing to the shearing effect of the Poiseuille fluid and the self-spontaneous collapse due to the insufficient magnetic constraint of the “massive” VPNS. Eventually, the maximum velocity of the VPNS can approach approximately 102 μm/s at a fluid impact of 400 μm/s. The velocity is numerically equivalent to approximately 340 times the body length of a single nanorobot (MNPs have a diameter of 300 nm) because the VPNS is composed of discrete nanoparticles with reconfigurability and deformability. By progressively increasing the pumped volume flow rate of the suspension (2 and 3 μl/min), the flow rate in the microfluidic channel increases correspondingly, but both maintain the classical parabolic distribution (Fig. S21C and D). The peak flow rate is approximately 1,200 μm/s at input volume flow rates up to 3 μl/min. Then, the VPNS only maintains upstream motility at the edge portions of the flow channel on both sides; however, it loses traction on the wall at the remaining locations and is eventually swept away by the water flow (Fig. S22).

From the side viewpoint, the VPNS basically shows 2 states: (a) a "rigid body" that maintains a certain height (h) with the wall and basically without deformation during the upstream process (Fig. 4E); (b) a "flexible body" in which the VPNS fits completely with the wall and creeps upstream (the schematic diagram is shown in Fig. 4D). The VPNS in the "flexible body" usually have a large cluster diameter, and they move upstream like white cells adhering to the wall, showing a peristaltic-like state, as shown in Fig. 4G, but due to the impingement of the shear flow, this flexible shape is usually unstable, and the large diameter of the VPNS will be broken into smaller individuals by the fluid. The motion of this rotational creep upstream process is simulated using COMSOL, as shown in Fig. 4F. On the contrary, the shape of VPNS in the "rigid body" state is more stable and can basically maintain the disc shape during the motion, which is more common for the upstream motion of VPNS.

Herein, we present a theoretical model of the VPNS rolling upstream along a microfluidic channel when a flow is applied. The micro swarm model is simplified to a thin-walled rigid disc of radius R and thickness t rotating about its normal. Observations and experiments show that upstream motion is possible when the linear velocity at the edge of the VPNS exceeds the fluid velocity. At an input magnetic field of 100 Hz, the rotational velocity of the micro swarm is considerably higher than the translational velocity, which implies the existence of a highly thin lubricated fluid layer at the contact area between the VPNS and the wall instead of pure rolling under dry friction. When the VPNS reaches the boundary layer, it is subject to multiple forces, as shown in Fig. S23. The low Reynolds number (*Re* = *ρ_f_U_f_* · *L_c_*/*μ* = 0.1099) and low swarm-mass-to-fluid-viscosity ratio under these experimental conditions enabled the dynamics to become overdamped; hence, the VPNS operating under a super-viscous fluid was in a forced equilibrium [21]. From a lateral view, the propulsive force *F_f_* due to wet friction being balanced by the viscous drag *F_D_* of the fluid along the translation direction of the VPNS, where *F_D_* = 6*πμR*(*U_p_* − *U_f_*)*f*_1_(*h*, *R*), considering the disc shape of the VPNS and the small gap against the wall *f*_1_(*h*, *R*). The additional term for the viscous drag is modified [33] to $F_{D}=2\pi \mu \left(U_{p}-U_{f}\right)t\sqrt{\frac{2R}{h}}$, where *U_p_* and *U_f_* denote the upstream velocity of the VPNS and the fluid velocity at the center of mass, respectively. The balanced equation for the forces in the horizontal direction is *F_f_* = *F_D_*. The forces experienced in the direction perpendicular to the propagation are more complex. At low Re, the time-reversal [5] motion of the VPNS produces no net propulsion, and it requires a break in the symmetry of the rotational motion, its structure, or the surrounding geometry [30]. The counterflow motion of VPNS in the experiment is mainly achieved by breaking the geometric symmetry during the rotational motion, close to the wall, and the irregular rheology. The Magnus effect (Fig. S24) indicates that both a rotating cylinder [34] and a parallel linear stream with an annular flow around a cylinder [35] produce a lateral lift on the cylinder, thereby causing it to move in a transverse direction; the schematic diagram of VPNS moving toward the wall is shown in Fig. 4A. The lift is due to the inconsistent flow rate between the 2 sides of the object, thus creating a lift from the high-pressure zone to the low-pressure zone. These forces are denoted by *F_RL_*and *F_SL_* in Fig. S23. In an application of this study, the lateral force *F_RL_* generated by the rotation drags the VPNS toward the pipe wall, whereas the lateral force *F_SL_* generated by the shear flow tends to push the VPNS away from the pipe wall. In 1965, Saffman et al. [26] demonstrated that the lift force generated by rotation is approximately one order of magnitude smaller than the shear, except when the particle rotates at a considerably faster speed than the shear. This precisely satisfies the conditions for the upstream mobility of the VPNS. Thus, the agreement between the experimental and numerical analysis results validates the theory. Figure 4B shows the side view in which the VPNS is moving toward the wall by the Magnus force, and this allows the VPNS to reach the low flow velocity boundary layer in the vessel, thus triggering upstream migration. Figure 4C shows the simulation of this process with the help of finite-element software, the VPNS rotating at high speed in the blood flow is considered as a rigid disc, and the lateral force generated by its rotation drags the VPNS to the vessel surface while generating vortices around it.

The force balance in the Y-direction is expressed as *F_SL_* + *F_N_* + *F_By_* = *F_RL_* + *G*, where *F_N_* is the supporting pressure provided by the fluid between the VPNS and the wall, and its product with the wet friction coefficient *μ_F_* is applied to the frictional force *F_f_* (i.e., *F_f_* = *μ_F_F_N_*). *F_By_* is the fluid buoyancy force, and *G* is the VPNS gravitational force, which is unavoidable because the “disc” can be dragged toward the wall by the aggregated magnetic particles. From the analysis of the horizontal and vertical dynamics, we derived an estimate for the upstream velocity of the VPNS, which is strongly dependent on its radius, thickness, rotational angular velocity, and pumped fluid (see Note S4 for details):

$$U_{p}=\frac{\text{πRtf}\left(M,S\right)\left(\rho_{P}-\rho_{f}\right)g}{84.63\mu t{\left(\frac{1}{\text{lnRes}}\right)}^{2}+2\mathrm{\pi \mu t}\sqrt{\frac{2R}{h}}-2{\pi \mu }_{F}\rho_{f}R^{2}\mathrm{t\Omega}}+U_{f},$$ (6)

where *f*(*M*, *S*) is a function of the material and surface with a unique fixed value [36]; *ρ_P_* and *ρ_f_* are the densities of the nanoparticle and fluid, respectively; *Res* indicates the shear Reynolds number, *Res* = *kR*^2^*ρ_f_*/*μ*; and k is defined to be the flow velocity gradient. The mathematical model established above was analyzed using Wolfram Mathematica (Wolfram Research), and a curve of the VPNS translational velocity versus diameter is shown in Fig. S25. The upward flattening tendency of the analytical solution and measured data (Fig. 4H) is reasonably satisfied by the VPNS rolling against the wall. The slight deviation in the curve fitting can be attributed to the following: (a) the upstream rolling VPNS is not perfectly circular, and it exhibits noticeable variations in collision and diameter distribution during the rheological process; (b) variations in the drag and wet friction coefficients at different locations in the flow channel; and (c) limitations of rigid-body features in predictive models for VPNS with flexible variability and reconfigurability. The simplified model that is established does not consider any shape and internal structural modifications in the VPNS rheology; however, it is sufficient to correctly describe the velocity characteristics of the micro swarm during upstream motion with tolerable deviations from the experimental values. The objective of this model is to demonstrate the interaction between the countercurrent propulsion of the VPNS and fluid flow.

For a single magnetic particle, its diameter range is 300 nm to 10 *μ*m. In this case, the particle diameter is independent of the magnetic field frequency, and the difference between particle velocity and fluid velocity is plotted at frequencies of 25, 250, and 2,500 Hz, as shown in Fig. S14. The difference is ^1^/_10_ of the fluid velocity at the same position, indicating that the particle velocity is mainly determined by the fluid velocity at the same position, which means that no matter how the diameter of the particle changes, it cannot move upstream. When the central flow velocity in the tube decreases from 2 m/s, its velocity distribution changes, as shown in Fig. S15A; as the flow velocity decreases to 0.1 m/s, the cluster can move upstream within the critical frequency (as shown in Fig. 4I). From the above steps, it can be concluded that it is desirable to reduce the flow velocity in order to achieve the upstream movement of the cluster. When setting the tube diameter as *D* = 2 cm, the phase diagram between frequency and velocity in the center of the tube can be obtained (Fig. S15B). Region II represents the effective region (e.g., *f* = 25 to 200 Hz), which means particles can swim upstream. Particles are not easy to form clusters in lower frequency (region I). On the other hand, with the increase of magnetic field frequency, the relaxation of clusters increases, indicating that clusters no longer rotate synchronously with the magnetic field. Moreover, clusters cannot swim upstream in region IV. For example, when setting magnetic field frequency *f* = 200 Hz, the critical central velocity equals 0.0836 m/s with the tube diameter *D* = 2 cm (Fig. S15C). We can also change the tube diameter to make clusters swim upstream. For instance, the critical diameter is 47.82 cm when *U_f_* = 2 m/s and *f* = 200 Hz. The critical diameter is meaningless for practical physiological conditions.

### Upstream movement and docking of VPNS in a multiphysiological environment

**Fig. 5.**
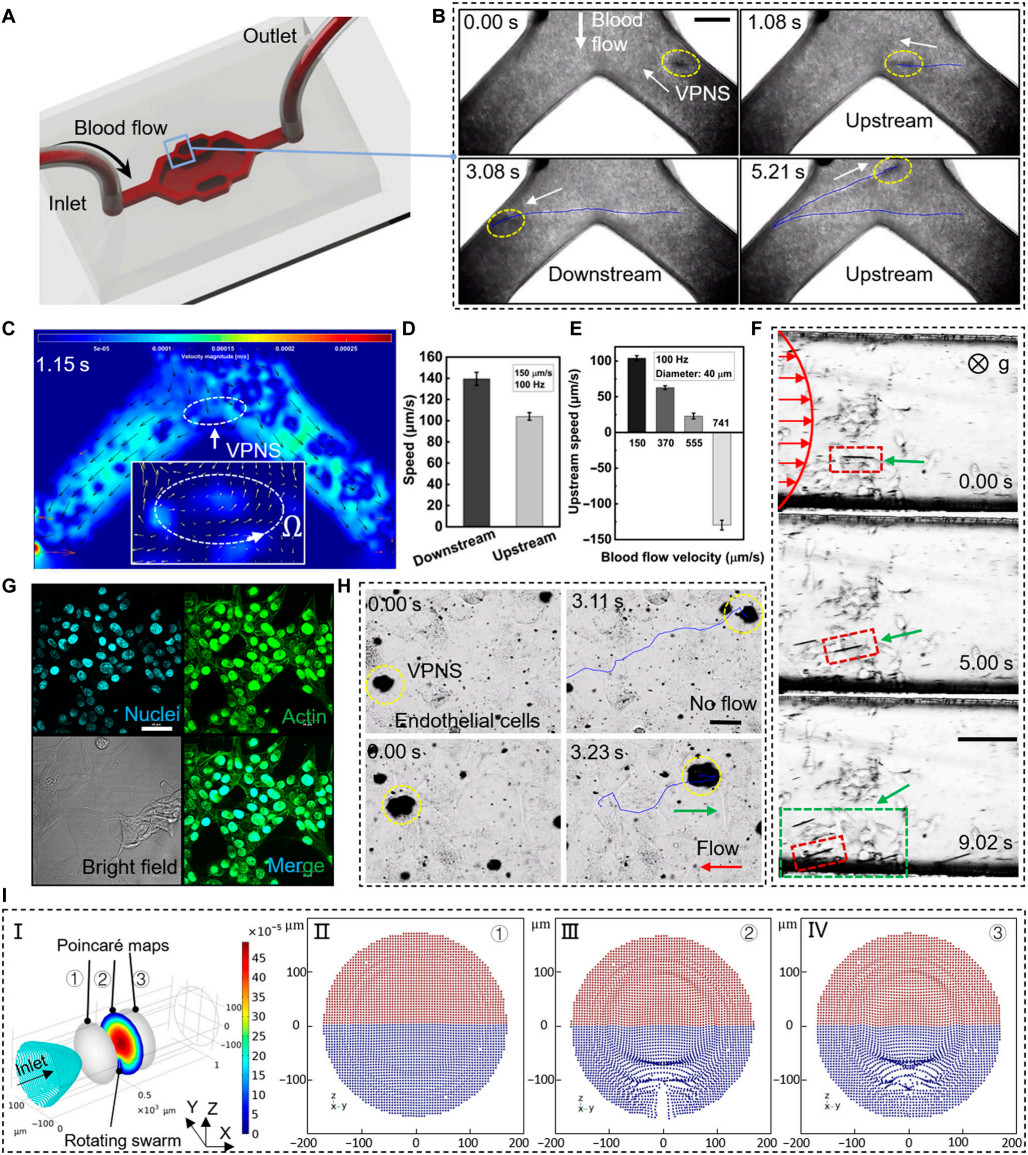
Upstream movement and docking of VPNS in a multiphysiological environment. (A) Diagram of pumping blood into a microfluidic chip. (B) Top view: Controllable movement of VPNS in blood flow, and the blue line is its trajectory. The scale bar is 100 μm. (C) Flow field analysis around VPNS in blood flow. The part surrounded by the white dashed line in the built-in subplot is VPNS. (D) The downstream and upstream velocity of VPNS. (E) Blood flow velocity versus upstream velocity of VPNS. (F) Upstream propulsion and targeted stopping of VPNS in microfluidic channels cultured with cancer cells. The black shuttle surrounded by the red dashed box is the designated VPNS, and in vitro-amplified 4T1 cells (white transparent blocks surrounded by green dotted lines) are spread on the bottom of the flow channel. The scale bar is 100 μm. (G) Endothelial cells under confocal microscopy, where blue fluorescence is the nucleus and green are actin. The scale bar is 100 μm. (H) Upstream movement of VPNS on endothelial cells and the magnetic field pitch angle of 40°. The scale bar is 50 μm. (I) Poincaré mapping of VPNS upstream motion in the blood stream. where II, III, and IV diagrams represent the distribution of blood cells in 3 of these sections, respectively.

In this section, we simulated multiple physiological environments in vitro, e.g., actual blood flow and vascular surfaces, and investigated the upstream motility of VPNS under these conditions. The greatest challenge for VPNS in navigating the vasculature is the fast-flowing blood and many adherent cells. We injected 6-fold diluted mouse blood into the microfluidic chips (Fig. 5A) and steered the VPNS population to advance and dock toward the lateral sides of the channel, as shown in Fig. 5B. When the motion time reaches 1.15 s, the VPNS moves to the bifurcation of the channel. According to our previous study [37], even in more complex simulated 3D vessels, VPNS can still freely travel between branches according to a preset trajectory. Analysis of the fluid state at this point indicates that, when the blood flows along the channel, a flow field disturbance appears around the VPNS corresponding to its rotation direction, as shown in Fig. 5C and its inset diagram. VPNS can achieve both downstream and upstream motion in a bloodstream with a flow rate of 150 μm/s and exhibits a velocity difference of approximately 40 μm/s, as shown in Fig. 5D. Given that the physical properties of blood are very different from those of water, the upstream velocity of VPNS at different blood flow velocities was monitored. The upstream velocity of the cluster tended to decrease as the blood flow velocity increased and eventually could not resist the blood flow and was washed away (Fig. 5E). The motivation for using 6-fold diluted blood was to enable relatively clear visualization of VPNS in in vitro experiments. The key parameters for upstream motion are blood flow velocity and viscosity, which have been aligned with whole blood in existing experiments. As for the issue of blood cell impaction, we performed finite-element simulations and verified that the generation of a "cell-free" boundary layer by high-speed rotation can effectively prevent VPNS from being scattered, as shown in Fig. 5I. Further experiments in whole blood and ultrasound imaging also confirmed the feasibility of this strategy.

Epithelial cells are grown in microfluidic channels to mimic the topography of the vascular surface. An upstream rolling VPNS controlled by a magnetic field provides movement on monolayer vascular endothelial cells, and it is capable of tumbling over the bulging nucleus obstacle with ease while maintaining rheology, eventually stopping at the tumor area. Figure 5G presents the staining results of endothelial cells using Hoechst 33258 and ghost pen cyclic peptides. Under confocal microscopy, the nucleus fluoresces blue, while actin, which forms the cytoskeleton, fluoresces green. Cells were propagated and expanded on a culture dish substrate and were grown using many adhesive proteins and receptors on the surface. This enabled a small number of MNPs to adhere to their surface, but it did not affect the upstream motility properties and reconfigurability of the VPNS that aggregated into swarms, as shown in Fig. 5H. Each VPNS formed in a flow environment is expected to travel along the direction of blood flow and achieve upstream rolling and braking hovers at the target location (e.g., the tumor region).

In the vasculature, the vessel wall represents the most favorable location for forced manipulation, which is evident in the migratory behavior of red blood cells under flow-stream conditions [38,39]. To test the propulsive capability of the VPNS under more realistic conditions, colonies of cancer cells were cultivated in the microfluidic chip channel and then pumped into the suspension of MNPs. The suspension of MNPs was pumped into the specialized flow channel using phosphate buffer saline (PBS) as a dispersant to maintain the osmotic pressure balance inside and outside the 4T1 cells. The VPNS was actuated by a rotating magnetic field of 100 Hz, and it could operate in a controlled upstream manner in the channel with epithelial cell, where the flow velocities exceed 300 μm/s. The orientation of VPNS was synchronized by modifying the yaw angle of the magnetic field. It formed an angle of approximately 160° with the direction of the flow. When the VPNS has an inclined angle relative to the fluid, it can yaw toward the side of the flow channel while maintaining upstream motility. During this process, the lateral navigation and counterflow states of the VPNS can be maintained in balance by fine-tuning the magnetic field yaw angle, as shown in Fig. 5F. The bottom surface of the flow channel was prelayered with a population of 4T1 cells, and the VPNS, framed by the red dashed line in the figure, docked to the cancer cell aggregation area on the left side of the channel within 9.02 s, which shows the ability of VPNS to moor in dynamic flow fields.

Rotating VPNS in flowing blood disturbs the blood flow, which provides the fundamental mechanism for the Magnus effect (simulations in Fig. 4C). To investigate rotational VPNS in flowing blood cells, a set of simulated erythrocytes (6 μm in diameter) were released from the vascular inlet, as shown in Fig. 5I(I). To visualize the movement of erythrocytes, the initial positions of all particles were divided into 2 groups on the basis of the Z-coordinate axis of the Poincaré map. The colors in the Poincaré map indicate the initial position of the particles (blue, Z < 0; red, Z > 0). Under flow conditions, the particles remain in the same position in the YZ plane close to the front of VPNS; see Fig. 5I(II). Some of the particles changed their initial position after contact with the vortex induced by the VPNS rotation but did not move to the other half plane, and a particle-free boundary layer appeared around the VPNS; see Fig. 5I(III) and (IV). Combined with the experimental results, it showed that the VPNS was robust enough to move upstream in the flowing blood cell population without being scattered by the cells and also greatly reduced cell adhesion.

### The photothermal killing of cancer cells by MNPs in vitro and in vivo experiments

**Fig. 6.**
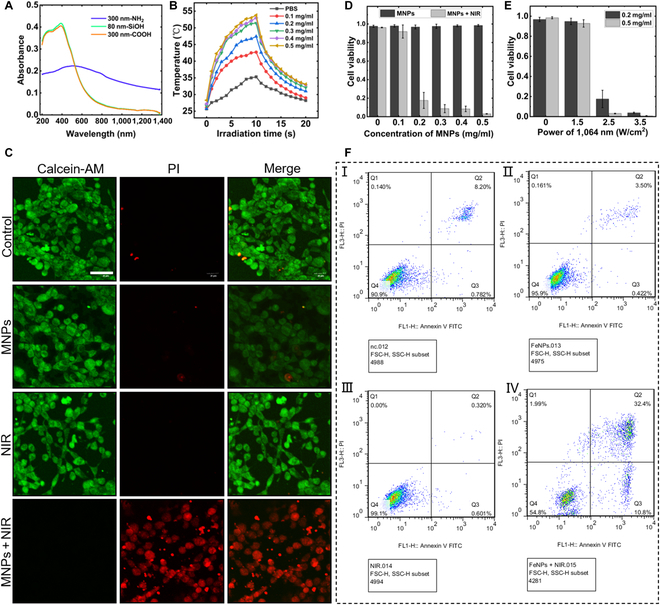
The photothermal killing of cancer cells by MNPs in vitro experiments. (A) Ultraviolet-vis-NIR absorption spectra of 3 types of the MNPs. (B) Temperature increase curves of VPNS suspensions with different concentrations under 1,064-nm NIR irradiation. (C) Laser scanning confocal micrographs of 4T1 cells costained with calcein-AM and PI, where live cells are stained with green fluorescence by calcein-AM, and dead cells are stained with red fluorescence by PI. The concentration of MNPs employed was 0.5 mg/ml, and the 1,064-nm NIR laser power density was adjusted to 2.5 W/cm^2^with a 3-min irradiation duration. The scale bar is 40 μm. (D) Cell viability at gradient concentrations of MNPs. (E) Association between the gradient power density of 1,064-nm NIR irradiation and cell viability. (F) FCM-led analysis of apoptosis in cancer cells. (I) Annexin V/PI staining only. (II) MNPs added only. (III) NIR-irradiated only. (IV) MNPs-NIR combined.

For tumor-targeted therapy, the applications of 1,064-nm near-infrared (NIR)-excited fluorescence imaging and photothermal therapy (PTT) are typically used to achieve high-resolution imaging and excellent deep-tissue treatment [40]. Wu and Jin [41] suggested that the enhancement of fluorescence and photothermal effects are mainly associated with the nature of nanoparticle surface ligands, which contain atoms (N, O) or moieties (–COOH, –NH_2_) with electron-providing properties. The NIR-visible-ultraviolet absorption spectra of MNPs with different particle sizes and functional groups were examined, and the absorbance curves are shown in Fig. 6A. The absorbance curves of 80-nm-diameter silica-based MNPs and 300-nm-diameter modified carboxyl MNP suspensions coincide under irradiation in the 200- to 1,400-nm spectrum, and they exhibit firm absorption peaks near 400 nm; however, absorbance is extremely low in the band beyond 800 nm. In contrast, MNP suspensions with 300-nm-diameter surface-modified amino groups have no clear absorption peaks in the entire wavelength band tested, and the difference between the absorbances at 808 and 1,064 nm is merely approximately 0.03, which is almost negligible.

Furthermore, irradiation at 1,064-nm NIR can induce a deeper penetration effect, which provides a new application scenario for PTT. Consequently, MNPs with 300-nm-diameter surface-modified amino groups were adopted for the experiment, which meets the requirements for safety and retention of nanoparticles in blood vessels [42]. We investigated the actual photothermal performance of MNPs; 5 different concentrations of nanoparticle suspensions were configured, 500 μl of each was collected and placed in petri dishes and irradiated with 1,064-nm NIR at a power density of 2.5 W/cm^2^ for 10 min. The laser was turned off for 10 min, and the temperature values of the suspensions with 20 min were recorded. The temperature variation curves are shown in Fig. 6B. Under the irradiation of the 1,064-nm NIR laser, the temperature of the suspension progressively increased; the higher the concentration of MNPs, the more significant the heating effect. The temperature of the MNP suspension with a concentration of 0.5 mg/ml was altered from 28.3 to 53 °C in 10 min, with a cumulative increase of 24.7 °C, which corresponds to the tumor killing temperature without damaging the viability of the normal cells surrounding the tumor [43]. The temperature of the suspensions with concentrations of 0.1, 0.2, 0.3, and 0.4 mg/ml increased by 16.4, 20.9, 24.6, and 24.9 °C, respectively. The control group was then placed in a PBS solution (dispersants) with a maximum temperature limited to 35 °C. The increase of 9.3 °C was not sufficient to eliminate the cells. Therefore, it is feasible to utilize the photothermal effect of MNPs to kill cancer cells.

We next evaluated the regional tumor photothermal effect of MNPs. 4T1 cells were subjected to uniform and monolayer expansion in vitro and were then used in multivariate orthogonal experiments to characterize the photothermal effect of MNPs on tumors. MNPs with a 0.5 mg/ml concentration was suspended in PBS solution and added to a normally growing 4T1 cell colony. They were coirradiated under a 1,064-nm NIR laser at a power density of 2.5 W/cm^2^ for 3 min after coincubation for 24 h. Cell viability was counted by costaining the cells with calcein-acetylmethoxy methyl ester (calcein-AM) and propidium iodide (PI), and the fluorescence microscopy images of confocal laser scanning are shown in Fig. 6C. By conducting 3 replicated experiments and calculating the statistics of the data, we obtained an average cell survival rate of 97.6% in the blank control group. The cell viability without NIR light irradiation, but with the addition of 0.1, 0.2, 0.3, 0.4, and 0.5 mg/ml MNP suspensions, all exceeded 97%. This indicates that MNPs demonstrated low cytotoxicity and high biocompatibility. Additionally, ferric tetroxide nanoparticles are the primary inorganic nanodrug carriers for clinically acceptable applications approved by the U.S. Food and Drug Administration. The measured temperature of the cell culture medium irradiated with just the 1,064-nm NIR laser was 28.9 °C (temperature increase of 3.2 °C), and it maintained a survival rate of 96.2% because the temperature was not sufficient to cause protein deformation and apoptosis [44,45]. At an MNP concentration of 0.2 mg/ml in combination with NIR irradiation, the cell viability declined significantly to 17.6%, and the parameter continued to decrease to 8.7%, 8.4%, and 3.1% as the concentration of the MNP suspension increased (Fig. 6D). This indicates that the thermogenic effect of PTT is closely associated with the concentration of MNPs. The effect of the 1,064-nm NIR laser power density on the survival rate of 4T1 cells was also investigated (see Fig. 6E). The 4T1 cell populations were added to a suspension of MNPs at a concentration of 0.5 mg/ml, and they were irradiated with the NIR laser at power densities of 1.5, 2.5, and 3.5 W/cm^2^ for 3 min. The resulting cell viabilities were 92.8%, 3.1%, and 0.6%, respectively, whereas MNPs at a concentration of 0.2 mg/ml produced cell viabilities of 94.8%, 17.6%, and 3.8% under the same laser irradiation conditions. This indicates that the PTT with MNPs is strongly dependent on the laser power density (see Fig. S26 and Note S5).

Given the imprecision of staining for counting dead and living cells, flow cytometry (FCM)-dominated apoptosis was applied to the experiment, and the results are shown in Fig. 6F. The cell survival rates are 90.9%, 95.9%, 99.1%, and 54.8% in the Annexin V/PI staining, MNPs, NIR-irradiated, and MNPs-NIR combined groups, respectively. The difference in the survival rates between these 2 approaches is mainly because calcein-AM/PI staining can only observe cells locally, whereas FCM can count all cells in the culture dish [37].

**Fig. 7.**
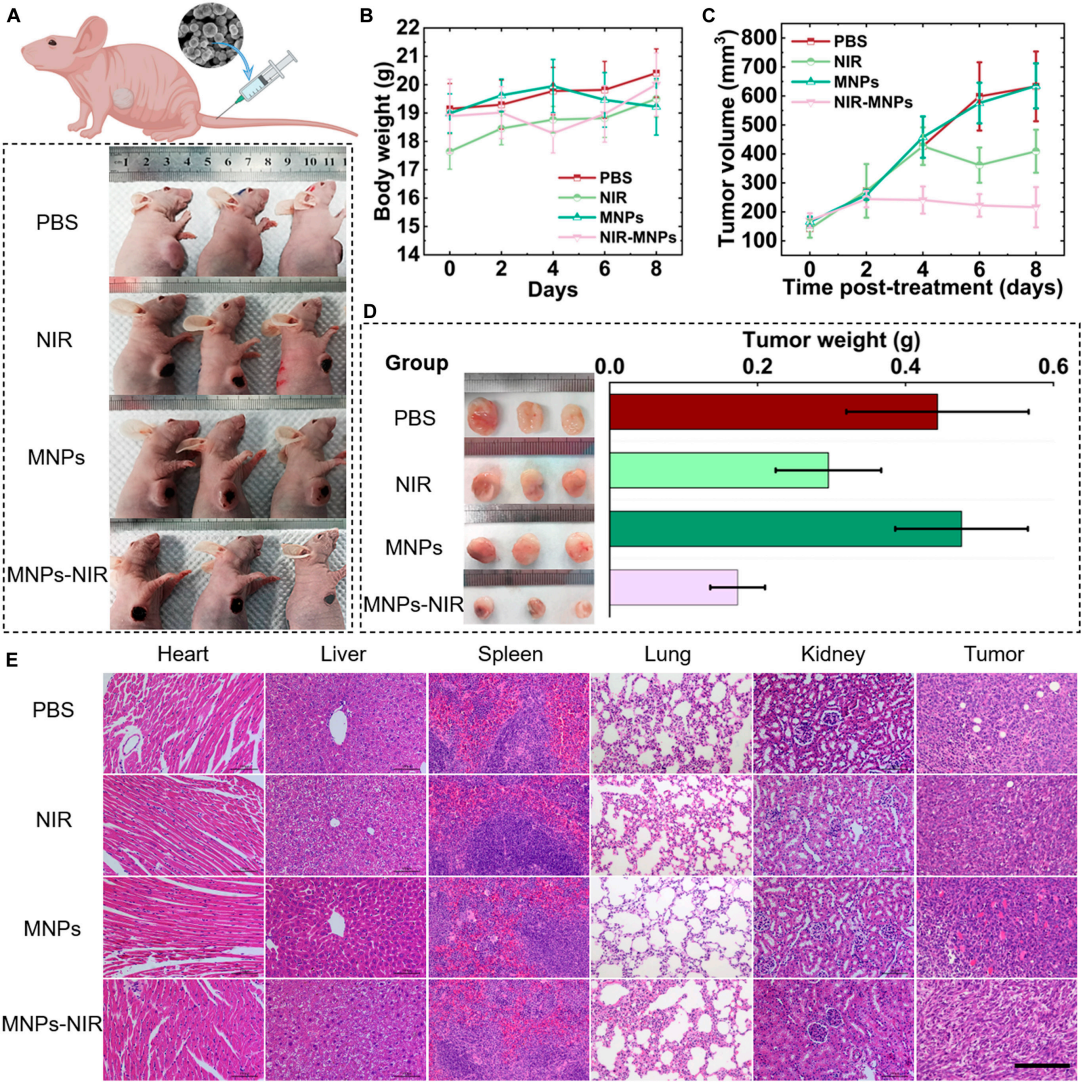
VPNS PTT for breast cancer in BALB/c nude mice. (A) Breast tumor-bearing BALB/c nude mice. (B) Body weight curves of 4 experimental nude mice during the treatment period. (C) Tumor volume curves of 4 experimental nude mice during the treatment period. (D) Tumor weight curves of 4 experimental nude mice during the treatment period. (E) Hematoxylin and eosin staining images of the main organs of nude mice after PTT. The scale bar is 200 μm.

Encouraged by the excellent in vitro PTT experiments achieved, we conducted in vivo tumor experiments on a mouse breast carcinoma tumor model. Four groups were included: (a) PBS, (b) NIR, (c) MNPs, (d) MNPs-NIR (*n* = 3 per group). Four groups of 4T1-bearing mice were injected with 0.2 ml of PBS or MNP suspension (PBS dispersion) at a concentration of 0.5 mg/ml through the tail vein, and then tumor location of the mice in the MNPs and MNPs-NIR groups were placed in a magnetic field with VPNS navigated to the tumor location, and after 2 h of magnetic field treatment, mice in the NIR and MNPs-NIR groups were treated with 1,064-nm laser irradiated (power density 2.5 w/cm^2^, 3 min). Such treatment procedure was repeated every 2 d for a 8-d course of treatment (Fig. 7A). To evaluate the anticancer effect of VPNS using the photothermal effect, we recorded the tumor growth rate (Fig. 7C). After one course of treatment, the tumor volumes of PBS, NIR, MNPs, and MNPs-NIR were 633.26, 409.32, 634.98, and 216.02 mm^3^, respectively. The results showed that the tumors in the PBS and MNP groups grew rapidly, indicating a failure to inhibit tumor growth by MNP enrichment alone. Laser irradiation could burn the tumor surface and slow down its proliferation to some extent, while the combination of MNPs and NIR had the best effect on tumor inhibition. After 8 d, tumors were removed and weighed, as shown in Fig. 7D, and the in vivo PTT results consistently showed that MNPs-NIR has excellent efficacy in the photothermal treatment of cancer. To confirm the safety of MNPs during PTT use, we performed histological analysis via standard histological techniques with hematoxylin and eosin staining. After 8 d of experiment, the mice were executed and the major organs were taken for histological analysis, and no visible tissue damage or adverse effects on major organs were observed, as shown in Fig. 7E. It has been preliminarily proved that MNPs has no obvious toxicity to animals at the test dose, and the experimental mice have neither significant weight loss (Fig. 7B) nor abnormal behavior observed. In conclusion, in addition to tumor elimination by PTT, MNPs has no obvious toxic and side effects on mice. Prussian blue staining of mice's major organs also showed that a certain amount of MNPs entered the tumor through the magnetic field, but due to the lack of blood vessels inside the tumor, such invasion only remained on the surface of the tumor but was sufficient to generate enough heat to cause tumor ablation (Fig. S27). Notably, the low-frequency alternating magnetic field in the experiment can induce a G1-phase delay in breast cancer cells but does not cause significant cell cycle perturbation, which is a property that allows the magnetic field to provide radiosensitization for the subsequent NIR thermotherapy [46].

## Discussion

It is well known that most tumors that grow beyond a small size need to generate a vascular supply to deliver nutrients to tumor cells that expand to distant sites [47]. The circulatory system may be an obvious route that can be utilized and navigated to approach the tumor [10]. To overcome the challenging environment in the vasculature, large amounts of red blood cells, sticky proteins, and harsh fluids, leukocytes in vivo utilize the low-flow zone and cell-free layer of the vessel wall to roll and propel themselves through the surface, which is considered the most feasible way to navigate the circulatory system [15]. In this study, we demonstrate a tether-free magnetic microrobotic swarm system that is inspired by a wild herring swarm and leukocytes. The VPNS exploits the no-slip boundary conditions of a Poiseuille flow near a vascular wall, and it combines with the actions of gravity, Saffman lift, and an external magnetic field to steer toward the wall, where it is subject to controlled upstream rolling. The VPNS constructed in this work constitutes a population of thousands of paramagnetic nanoparticles assembled in response to a magnetic field, and the assembly and navigation are highly robust and advantageous. For example, the cumbersome steps, complicated fabrication methods, and prohibitive cost of some structured prefabricated microrobots are important impediments to their rapid mass production on a large scale. By contrast, we used commercially sold paramagnetic nanoparticles with diameters in the range of 80 to 300 nm, which are used for Food and Drug Administration-approved inorganic nanomedicines with biocompatible and magnetically responsive properties. Additionally, only the magnetic field is required to achieve MNP assembly, traction wall attachment, and active upstream functions.

The dynamically assembled VPNS has a flexible leukocyte-like variable structure that adheres to the vessel wall, actively propels flow at relative speeds of up to 100 μm/s, and can precisely control navigation and mooring in complex process configurations of physiological blood flow. In addition, the dissociative and recombination ability of VPNS reduces the risk of intravascular thrombosis. In addition to the kinematic properties of the magnetic microrobot, the success of the upstream motion depends on the applied flow rate and shear stress. The physiological level of blood flow illustrated here corresponds to the capillaries and small veins; more importantly, it is associated with the majority of the supply of blood flow in solid tumors, which are also the main areas of intimate contact with the tumor and leukocyte extravasation [48]. Higher flow rates and shear stresses can be countered by increasing the frequency and strength of the magnetic field. The lower blood flow velocity (300 to 1,000 μm/s) and wall shear stress [49] (2.5 dyn/cm^2^) in the small postcapillary veins caused the vascular endothelial cells to exhibit a relatively minor aspect ratio [50]. The endothelial cell nuclei bulge creates a barrier height of approximately 4 μm, which experimentally verified that the VPNS could be rolled and propelled through a dynamic flow field of densely packed cells while maintaining flow state variability. Along with traversing the rugged cell-layered wall, it is necessary to test the upstream migration of the VPNS against gravity in the 3D structure of the vasculature. When the inclination angle of the plane of continuous motion is greater than 0°, the microrobot often requires the assistance of external energy to overcome gravity, such as magnetic forces from vertical walls [51] or ultrasonic radiation [27]. Previous studies have demonstrated that our constructed VPNS possesses a combination of liquid and solid behavior, and it can accommodate stairs and gaps that are equivalent to its height [52]. The corresponding tumor-killing method must be implemented when the VPNS converges along the blood vessels and is actively moored in the tumor region. Compared with widely used 808-nm NIR light, 1,064-nm NIR improves the penetration depth and brings a more intense PTT and fluorescence imaging deeper in the tissue [40], and in vitro-simulated cancer-cell-killing experiments have also verified its reliability.

We believe that the proposed method is feasible for further in vivo experiments. In a magnetic field environment, where no other ferromagnetic material is present in the organism, magnetic shielding will not occur, and no significant field-strength decay is expected. Under the current experimental conditions, the blood flow velocity that VPNS can withstand is limited to all capillaries and some small veins. Theoretically, the following measures could increase its upstream velocity and expand its application: (a) Increase the magnetic field strength, using a magnetic field coupled to a robotic arm to improve upstream capability and control range. (b) Adapting the boundary layer of the vessel wall as close as possible. (c) Increase the magnetic field frequency in a certain range: 25 to 200 Hz. In vivo real-time imaging of VPNS has been a challenge in the microrobotics field [53]; however, recent studies have shown that photoacoustic imaging [54] and magnetic particle imaging [55] are being applied to real-time tracking of tissues and viscera in mouse models, which may provide practical real-time tracking tools for our future research. Our future research will focus on adding in vivo experimental imaging modalities for real-time VPNS tracking and navigation, as well as quantitative assessment of nanoparticles reaching the tumor to determine drug utilization rate. In contrast, the research in this manuscript is primarily aimed at revealing the principles of clustered upstream motility behavior of VPNS, achieving precise motion control and physical modeling of VPNS, and preliminary validation of targeting capabilities.

## Materials and Methods

### Pre-experimental treatment of MNPs

The paramagnetic nanoparticles used in the experiments were purchased from Bionano New Material Science, Taizhou, China. The concentration of the raw solution was standardized to 10 mg/ml, and the solution was distributed in deionized water. After the desired concentration was configured with the PBS solution, the suspension was oscillated in an ultrasonic cleaner for 5 to 10 min to obtain better dispersion before performing the magnetic actuation experiments.

### Microfluidic chip fabrication

The molds for the microchips were manufactured using high-quality 3D printers (NanoArch P140, BMFPrecision, Shenzhen, China) with photosensitive resin material. The microfluidic channels were realized using PDMS molding technology. First, the organic elastic matrix of a Sylgard 184 (Dow Corning, Midland, MI, USA) was mixed with a Sylgard 184 curing agent at a mass ratio of 10:1. It was mixed thoroughly and put into a vacuum pump for 30 min to eliminate the internal air bubbles. Then, the surface of the mold was processed with 1H,1H,2H,2H-perfluorooctyltrichlorosilane (Sigma-Aldrich, USA), which was used as a hydrophobic treatment to utilize the molding easily. Next, the PDMS mixture was poured into the bottom-fixed mold and then put in a hot oven above 60 °C for more than 3 h to complete the curing. Finally, the curing layer was separated from the mold and perforated with exit and entrance holes. The final microchannel structures and glass slides were treated with plasma (YZD08-2C, SAOT, Beijing, China) for 30 s to form surface covalent bonding.

### 4T1 cell culture

The 4T1, was sourced from the National Infrastructure of Cell Line Resource. It was maintained in a Roswell Park Memorial Institute medium (RPMI 1640, Gibco, Amarillo, TX, USA), which contained 10% fetal bovine serum (Vistech, New Zealand), and it was cultured at 37 °C with a 5% CO_2_ concentration. For collection, cells were dissociated from the bottom surface of the culture dish using 0.1% trypsin-ETDA (Gibco, Amarillo, TX, USA) and centrifuged at 600 G for 3 min. The supernatant was discarded, and a new culture medium was added to resuspend the cell pellet and transplant it into the experimental environment. The covalently bonded chips were sterilized via autoclaving to culture cells in the fabricated microfluidic chips. Subsequently, poly-lysine (Coolaber, Beijing, China) was injected into the channel in a sterile table and discarded after 5 min of filling to enhance cell apposition. Then, the required cell lines were inducted after the chip was dried, keeping appropriate droplets at the inlet and outlet of the flow channel to prevent excessive evaporation. Finally, the cells were placed in a humid environment to expand and propagate.

### Mouse blood acquisition and preservation

Blood was obtained from 8-week-old female Balb/c mice weighing 18 to 20 g (Sipeifu Biotechnology Co., LTD, Beijing, China). Cardiac blood samples were obtained and rapidly preserved in sodium citrate anticoagulant (Leagene Biotechnology Co., LTD, Beijing, China), maintaining a volume ratio of 1:9 between the components.

### Magnetic field control

The VPNS was actuated with 6 customized electromagnetic coils (4 XY coils and 2 Z coils), and above the Z coils, a charge-coupled device camera (HT2000) with a microscope head (Olympus, Tokyo, Japan) was used to acquire images. Specifically, the VPNS is driven in 2 dimensions by applying a user-programmed current to the electromagnetic coils to generate the desired customized magnetic field.

### Active targeting and upstream locomotion of VPNS

For experiments in the static flow field, a 5-μl drop of the MNP suspension was dropped onto a glass slide with a clean surface. Another layer of glass was used to cover the surface, but the glass layers were separated with double-sided tape to maintain a spacing of approximately 100 μm between them. For experiments with kinetic flow fields, the suspension was pipetted into a 1-ml syringe with a plastic hose attached at the outlet to be introduced into the specialized microfluidic channel. The syringe was then fixed to a high-resolution syringe pump (PHD Ultra Nanomite, Harvard, Cambridge, MA, USA) to set the desired parameters for the experiment.

### Cell staining and characterization

The cells were stained with dual dyes calcein-AM and PI (Solarbio Life Sciences, Beijing, China). First, the 10× assay buffer, calcein-AM (2 mM), and PI (1.5 mM) were removed from the −20 °C freezer to thaw and rewarm by avoiding light for 30 min. Then, the appropriate amounts of calcein-AM and PI were extracted according to the single-dosage amount (300 μl per experiment well) and diluted 100 and 33.3 times with PBS solution, respectively, to obtain the preset dye solution (10× calcein-AM and PI). Finally, 10× assay buffer, 10× calcein-AM, 10× PI, and deionized water were mixed uniformly at a volume ratio of 1:1:1:7 to obtain the staining working solution. The culture medium containing MNPs in the 4T1 cells was discarded, and the staining solution was added slowly after washing twice with PBS solution while avoiding light. The stained cells were observed under a laser scanning confocal microscope (FV3000, Olympus, Tokyo, Japan). The green and red fluorescence excitation wavelengths were adapted to approximately 490 and 535 nm, respectively, to distinguish between live and dead cells in the visual field.

### Computational fluid dynamics simulation

The finite-element analysis software COMSOL Multiphysics 5.5 was used to estimate the flow velocity, pressure distribution, and forces acting on the VPNS in the microfluidic channel under static and flow conditions. The simulations were performed in a 2-dimensional plane and 3D space to solve the Navier–Stokes equations for a micro disc placed in a rectangular channel fed with deionized water at a constant volume flow. A no-slip boundary is considered in the simulation model, and the VPNS was simulated as a thin-walled disk with a diameter of 60 μm and a thickness of 2 μm spinning at a frequency of 10 to 100 Hz with a flow input of 1 μl/min. The velocity and pressure fields were determined using a peristaltic flow module.

### Statistical information

All quantitative values in the experimental results were expressed as a mean ± standard deviation. Statistical analysis was performed using the Student *t* test, the statistical significance was set at a 95% confidence level (*P* < 0.05), and each data acquisition experiment was repeated at least 3 times.

## Data Availability

Data is avaliable on request.

## Supplementary Materials

Supplementary 1 Note S1. Dynamics of single particle. Note S2. Interpretation of the transformation from MNP suspension to vortex. Note S3. Flow field distribution model for microfluidic flow channels with a square cross-section. Note S4. Dynamics model of simplified VPNS upstream rolling in the Poiseuille flow. Note S5. Manipulation of clusters. Fig. S1. Electron micrographs and magnetic characterization of MNPs. Fig. S2. Force on a single magnetic particle in a magnetic field. Fig. S3. Vibration state of the nonuniform oscillators system. Fig. S4. Particle rotation frequency and magnetic field frequency. Fig. S5. Analysis of magnetic particles near the boundary. Fig. S6. Viscous force and fluid drag force vary with distance from the bottom. Fig. S7. Simulation of local vortices generated by short-chain rotation. Fig. S8. Analysis of particle chain stability. Fig. S9. Fluid analysis during the VPNS merging. Fig. S10. Forces on the particle at the edge. Fig. S11. Comparison of 2 models. Fig. S12. Force analysis of VPNS in a dynamic flow field. Fig. S13. Analysis of flow field velocity and cluster velocity versus frequency. Fig. S14. Translation velocity of a single particle. Fig. S15. Analysis of the upstream motility of VPNS in high-speed blood flow. Fig. S16. The main body of the magnetic manipulation platform. Fig. S17. VPNS wall-mounted rotational simulation. Fig. S18. Snapshot of the upstream rolling VPNS. Fig. S19. Simulation analysis of VPNS viscous drag versus rolling pitch angle. Fig. S20. Fabrication of the microfluidic channel. Fig. S21. Flow rate profile at higher volume flow rates. Fig. S22. Upstream motility of VPNS at higher flow rates. Fig. S23. Schematic diagram of the force analysis of VPNS moving upstream in the Poiseuille flow. Fig. S24. Numerical and analytical analysis of the Magnus effect. Fig. S25. Analytical relationship between the VPNS upstream velocity and diameter. Fig. S26. The photothermal effect of MNPs induces apoptosis in 4T1 cells via 1,064-nm NIR irradiation for 3 min. Fig. S27. Prussian blue staining images of major organs in PBS and MNPs-NIR groups. Table S1. Parameters used in the theoretical analysis. References [56–60]
