# Supplementary material for: Reconfigurable Vortex-like Paramagnetic Nanoparticle Swarm with Upstream Motility and High Body-length Ratio Velocity
Source: Research (Wash D C). 2023 Mar 27;6:0088. doi: 10.34133/research.0088 (PMC10042322; doi:10.34133/research.0088)
Supplement: Supplementary 1 — Note S1. Dynamics of single particle. Note S2. Interpretation of the transformation from MNP suspension to vortex. Note S3. Flow field distribution model for microfluidic flow channels with a square cross-section. Note S4. Dynamics model of simplified VPNS upstream rolling in the Poiseuille flow. Note S5. Manipulation of clusters. Fig. S1. Electron micrographs and magnetic characterization of MNPs. Fig. S2. Force on a single magnetic particle in a magnetic field. Fig. S3. Vibration state of the nonuniform oscillators system. Fig. S4. Particle rotation frequency and magnetic field frequency. Fig. S5. Analysis of magnetic particles near the boundary. Fig. S6. Viscous force and fluid drag force vary with distance from the bottom. Fig. S7. Simulation of local vortices generated by short-chain rotation. Fig. S8. Analysis of particle chain stability. Fig. S9. Fluid analysis during the VPNS merging. Fig. S10. Forces on the particle at the edge. Fig. S11. Comparison of 2 models. Fig. S12. Force analysis of VPNS in a dynamic flow field. Fig. S13. Analysis of flow field velocity and cluster velocity versus frequency. Fig. S14. Translation velocity of a single particle. Fig. S15. Analysis of the upstream motility of VPNS in high-speed blood flow. Fig. S16. The main body of the magnetic manipulation platform. Fig. S17. VPNS wall-mounted rotational simulation. Fig. S18. Snapshot of the upstream rolling VPNS. Fig. S19. Simulation analysis of VPNS viscous drag versus rolling pitch angle. Fig. S20. Fabrication of the microfluidic channel. Fig. S21. Flow rate profile at higher volume flow rates. Fig. S22. Upstream motility of VPNS at higher flow rates. Fig. S23. Schematic diagram of the force analysis of VPNS moving upstream in the Poiseuille flow. Fig. S24. Numerical and analytical analysis of the Magnus effect. Fig. S25. Analytical relationship between the VPNS upstream velocity and diameter. Fig. S26. The photothermal effect of MNPs induces apoptosis in 4T1 cells via 1,064-nm NIR [file research.0088.f1.pdf]

Manuscript Template

Title: Reconfigurable vortex-like paramagnetic nanoparticle swarm with upstream motility and high body-length ratio velocity

SUPPLEMENTARY MATERIALS

Note S1. Dynamics of single particle

The majority of ferric tetroxide magnetic nanoparticles that constitute VPNSs have an average diameter of 300 nm, and they are modified on the surface using amino groups to improve the monodispersity of the magnetic nanoparticles in a colloidal system. Photographs of MNPs taken using scanning electron microscopy are shown in Fig. S1A. Most MNPs have a diameter of approximately 300 nm at a magnification of 10 000×, and they retain well-defined sphericity and mono-dispersity. We used a vibrating sample magnetometer (VSM 7404, Lake Shore Cryotronics Inc., Westerville, OH, USA) to examine the endogenous magnetic properties of MNPs, such as magnetization strength, coercivity, and residual magnetism. The hysteresis lines obtained from these tests are shown in Fig. S1B. It can be observed from Fig. S1B(I) that the magnetic moment of MNPs shows an "S"-shaped trend when the external magnetic field varies between -20 and 20 Oe.

Moreover, the hysteresis line is exceptionally narrow, which indicates that the magnetic permeability of MNPs is significant, whereas the coercivity and residual magnetism are extremely small (Fig. S1B(II)). The magnetic moment of the MNPs was generated with the loading of the magnetic field, and the residual magnetism was only approximately 2 emu/g after the unloading of the magnetic field. The saturation magnetization intensity of the MNPs reached 58.55 emu/g, thereby demonstrating the superparamagnetic properties of the MNPs used in this study.

Critical frequency When subject to a rotational magnetic field, a single particle is magnetized and rotated by magnetic force moment in unbound fluid (Fig. S2). And the rotation dynamics equation in the fluid is given:

$$I\ddot{\theta} = -\gamma\dot{\theta} + mBsin(\omega_B t - \theta) + T(t) \tag{1}$$

Where I is inertia moment, $\gamma = 8\pi\mu a^3$ is the rotational drag coefficient of a spherical particle with radius a in the fluid whose viscosity is μ. And m, ω_B, T represents the particle's magnetic moment, the angular velocity of the magnetic field, and Brownian moment respectively[52].

Generally, the Brownian moment can be ignored because the driving moment of the external magnetic field is much larger than it. And the Reynolds number derived from the ratio of inertia force and resistance of the rotating spherical particle:

$$R_e = \frac{a^2\rho\dot{\theta}}{\mu} = \frac{a^2\dot{\theta}}{\upsilon} \tag{2}$$

Where ρ is the fluid density, μ is hydrodynamic viscosity, υ is kinematic viscosity. For example, when a particle with a radius of 40μm is rotating at an angular velocity of 100 rad/s in water with a kinematic viscosity of 1.007cSt (20℃), the Reynolds number is 2.56×10^{-10}, which conforms to $Re \ll 1$, which means the influence of inertia on particle motion can be ignored. Thus, equation (1) can be simplified as:

$$-\gamma\dot{\theta} + mBsin(\omega_B t - \theta) = 0 \tag{3}$$

By substituting variables, equation (3) can be reduced to:

$$\dot{\varphi} = \frac{\omega_B}{\omega_c} - sin\varphi \tag{4}$$

Where $\varphi = \omega_B t - \theta$, $\tau = \omega_c t$, $\omega_c = mB/\gamma$, $\dot{\varphi} = d\varphi/d\tau$. Equation (4) formally conforms to nonuniform oscillators. Fig. S3 shows the vibration state of the nonuniform oscillators system as the ratio ω_B/ω_c changes.

Assuming that transient decay to zero as well as the system reaches steady-state. When $\omega_B/\omega_c < 1$, equation (4) approaches to the fixed point $\varphi = arcsin\,(\omega_B/\omega_c)$, therefore in a stable state with $\dot{\varphi}$=0. When$\omega_B/\omega_c > 1$, average velocity within a period is calculated:

$$\langle\dot{\varphi}\rangle = \frac{1}{T}\int_0^T \frac{d\varphi}{d\tau}d\tau = \frac{1}{T}\int_0^{2\pi} d\varphi = \frac{2\pi}{T} = \omega_B\sqrt{1-(\omega_c/\omega_B)^2},\ \ \omega_B > \omega_c \tag{5}$$

The vibration period is as follows:

$$\mathrm{T} = \int d\tau = \frac{1}{\omega_c}\int_0^{2\pi}\frac{d\tau}{d\varphi}d\varphi = \int_0^{2\pi}\frac{d\varphi}{\omega_B - \omega_c sin\varphi} = \frac{2\pi}{\sqrt{\omega_B^2 - \omega_c^2}} \tag{6}$$

Thus, the average angular velocity of the particle is:

$$\omega = \langle\dot{\theta}\rangle = \begin{cases} \omega_B & \omega_B < \omega_c \\ \omega_B\left[1-\sqrt{1-(\omega_c/\omega_B)^2}\right] & \omega_B > \omega_c \end{cases} \tag{7}$$

Where ω_c is the critical angular velocity of a particle, corresponding to the critical frequency is f_c (Fig. S4).

The above theory can be summarized as follows: regardless of inertia and Brownian forces, the rotation of a spherical magnetic particle is controlled by the interaction of magnetic force moment and viscous moment and exhibits two different modes of motion: synchronous and asynchronous rotation.

When $\omega_B < \omega_c$, the phase lag between the particle and the magnetic field is constant because the particle rotates synchronously with the magnetic field because of the balance between magnetic force moment and vicious moment.

When $\omega_B > \omega_c$, the binding of the particle by the viscous force exerted by the surrounding liquid environment exceeds magnetic driving force, and the phase lag between particle and

magnetic field increases with the increase of frequency, indicating that particle no longer rotates synchronously with the magnetic field and its angular velocity decreases with the increase of magnetic field frequency.

The Mason number is defined as the dimensionless ratio of the magnetization to the viscous force of the particles, that is, $M_n = \frac{\mu\omega}{\mu_0 \chi_p H^2} \frac{n^3}{(n-1)\left[\ln\left(\frac{n}{2}\right)+\frac{2.4}{n}\right]}$. When there are only two particles, the time t is defined as the shear time required for one particle to migrate under the influence of the other, and ω^{-1} denotes the shear time of a particle moving at an angular frequency ω. When $t < \omega^{-1}$ ($M_n < 1$), the magnetic force acting on the particles exceeds the viscous force; hence, the particles aggregate into short chains. As M_n increases, the magnetic force operating on the particles tends to decrease, and chains of a shorter length are formed, especially when $t > \omega^{-1}$ ($M_n < 1$). The shearing time of the particles is less than the time required for the particles to move under the magnetic field, which results in no stable chains being established. Neglecting the magnetization relaxation effect of MNPs, the particles' angular frequency ω of the particles is approximately equal to the magnetic field rotation frequency, and Mn is described as the most relevant function of ω and n (the number of magnetic fields particles). The specific number of particles in the short-chain was measured from 20 to 40, and the median n = 30 was selected for the analytical treatment. The Mason number and magnetic field frequency curves are shown in Fig. S8A. Theoretically, if the magnetic field frequency exceeds approximately 20 Hz, the particle chain is destabilized, consistent with the experimental results (Fig. S8B).

Wall effect When a spherical particle on the bottom surface rolls, it moves horizontally because of the role of horizontal flow in a particle's physical imbalance; the force analysis is shown in Fig. SA. Furthermore, velocity distribution function P(V) as shown in Fig. S5B around 4 mm/s at peak. The size of the peak depends on the driving magnetic field frequency. Moves on the non-slip boundary, particle's speed limit is $V_0 = \omega a$. Fig. S5C shows that the rolling velocity distribution of particles varies with frequency, and the velocity distributes around 0.2V_0~ 0.3 V_0.

Two main factors can cause velocity to decline. First, fluid can act as a lubricant, causing partial slip between particle and boundary. Second, a rotating particle moving horizontally near the solid surface experience a lift force F_L, which is opposite to the direction of gravity. According to Stokes' law, it can be deduced that the lift force of rotating particle is:

$$F_L = \pi\rho a^3 \omega V \tag{8}$$

Where V is particle translation velocity. When F_L is equal to gravity$G = 4/3\pi\rho_p a^3 g$, the particle has no contact with the bottom surface.

Using $V = V_0$, the critical condition ($F_L/G = 3\rho a\omega^2/4\rho_p g > 1$) for the particle to escape from the bottom surface can be obtained. Aranson et al.[53] observed that for a nickel particle with a radius of 60~100 µm ($\rho_p/\rho \approx 9$), the lift force will exceed gravity while $f_B \approx$ 60~100 Hz, which means that the particle cannot contact the bottom for a long time above this frequency. Accordingly, it should move parallel to the surface at speed less than V_0.

Fang et al.[54] research shows that when a particle rotates near the boundary, the horizontal viscous force of the particle is:

$$F_u = \pi\mu a^2 \omega f_u \tag{9}$$

Where:

$$f_u = \frac{3}{4}\left(\frac{a}{h}\right)^4 (1 - \frac{3a}{8h}) \tag{10}$$

When a particle moves horizontally, the drag force in the horizontal is:

$$F_d = 6\pi\mu a V f_d \tag{11}$$

Where:

$$f_d = 1 - \frac{9}{16}\left(\frac{a}{h}\right) + \frac{1}{8}\left(\frac{a}{h}\right)^3 - \frac{45}{156}\left(\frac{a}{h}\right)^4 - \frac{1}{16}\left(\frac{a}{h}\right)^5 \tag{12}$$

In addition, the bottom surface of a particle is subjected to the friction of the fluid.

$$F_f = \mu_f N \tag{13}$$

Where μ_f is the fluid friction coefficient.

The curves of the magnetic particle viscous force F_u and fluid drag force F_d vary with distance from the bottom surface are shown in Fig. S6.

Note S2. Interpretation of the transformation from MNP suspension to vortex

Under the action of the external magnetic field, the spinning particle generates a small vortex field with itself as the vortex core. Spin-induced hydrodynamic interaction and magnetic dipole force attract nearby particles to get close to each other, while the short-range repulsion prevents the particles from getting too close together. As more particles continue to gather, a vortex is formed.

2.1 Emergence of vortex

The Formation Process Vortex exerts long-term attraction on adjacent systems as a dynamic gathering system. When two vortices are far apart, that is, the center distance d of two vortices is greater than radius R of a vortex, vortices rotate slowly. When R/d exceeds a particular critical value, the attraction between vortices leads to vortex fusion. The phenomenon of vortex merging largely depends on the initial value of (R/d). When the ratio is lower than 0.3, the primary vortex forms a large stable vortex by swallowing small vortices one by one along a spiral trajectory.

This process can be summarized into three stages exchange, fusion, and stabilization, as shown in Fig. S9.

First, once two vortices are close enough, the vortices move towards each other driven by vortex advection (long-range attraction interaction), and the two closest parts of vortices begin to exchange members.

Then, when two vortices contact each other, all members enter the fusion zone, and an irregularly shaped vortex forms quickly. Meanwhile, the vorticity field merges into one through the diffusion process. Next, the vortex merges into an ellipse and spits out two strong vortex filaments.

Eventually, a stable circular symmetrical vortex is formed, fusing all members of these two vortices, while filaments gradually curl up and dissipate around the vortex core.

Surface fraction Since density-dependent diffusion in non-equilibrium systems leads to phase separation and a large number of fluctuations, and the surface fraction has a significant effect on the dynamic assembly of vortices. Research shows that in a given period, the surface fraction is lower levels (φ ≈ 2.08%) observed only a few small vortexes (less than 5 particles), and big vortex present at higher levels (φ ≈ 11.99%)[25].

Furthermore, the critical value R/d can estimate the minimum surface fraction required for vortex formation[27].

2.2 Parameter of collective dynamics

Diameter Magnetic force between two magnetic particles can be expressed as

$$\boldsymbol{F_m} = \frac{3\mu_0}{4\pi r_{12}^5}\left[(\boldsymbol{m_1}\cdot\boldsymbol{r_{12}})\boldsymbol{m_2} + (\boldsymbol{m_2}\cdot\boldsymbol{r_{12}})\boldsymbol{m_1} + (\boldsymbol{m_1}\cdot\boldsymbol{m_2})\boldsymbol{r_{12}} - 5\frac{(\boldsymbol{m_1}\cdot\boldsymbol{r_{12}})(\boldsymbol{m_2}\cdot\boldsymbol{r_{12}})}{r_{12}^2}\boldsymbol{r_{12}}\right] \quad (14)$$

Where vector $\boldsymbol{m_1}$ and $\boldsymbol{m_2}$ are the magnetic moment of particle 1 and particle 2, vector $\boldsymbol{r_{12}}$ is the center distance between particles 1 and 2, μ_0 is vacuum permeability[55].

Model-1 When the particle cluster is regarded as a rigid disk (Fig. S10), the particle at edge conforms to the law of circular motion, and the component of magnetic force along the central line ($\boldsymbol{F_m^R}$) provides the centripetal force.

$$\boldsymbol{F_m^R} = m_p\Omega^2\boldsymbol{r_{12}} \quad (15)$$

After a certain simplification of Equation (15) ($r_{12} \approx R$), the cluster radius varied with its rotational speed can be obtained.

$$R = \sqrt[5]{\frac{3\mu_0 k_m^R}{m_p\Omega^2}} \quad (16)$$

Where m_p is mass of particle 2, and $\boldsymbol{F_m^R}$, $\boldsymbol{k_m^R}$ represent radial component along the central line, $\boldsymbol{k}$ is the sum of the vector product related to the magnetic moment.

$$\boldsymbol{k} = \frac{(\boldsymbol{m}_1 \cdot \boldsymbol{r}_{12})\boldsymbol{m}_2 + (\boldsymbol{m}_2 \cdot \boldsymbol{r}_{12})\boldsymbol{m}_1 + (\boldsymbol{m}_1 \cdot \boldsymbol{m}_2)\boldsymbol{r}_{12} - 5\frac{(\boldsymbol{m}_1 \cdot \boldsymbol{r}_{12})(\boldsymbol{m}_2 \cdot \boldsymbol{r}_{12})}{r_{12}^2}\boldsymbol{r}_{12}}{r_{12}} \tag{17}$$

If magnetic moments of particle 1 and particle 2 are assumed to be parallel, which means the direction of magnetic force is along the centerline, then the equation (17) can be simplified as $k = -2m_1m_2$, and its radial component is itself. Total number of particles in the cluster is $m_1 = (R^2/a^2)m_2$. Thus, equation (16) can be further simplified to:

$$R = \sqrt[3]{\frac{9\mu_0 m_2^2}{8\pi\rho_p a^5 \Omega^2}} \tag{18}$$

Equation (18) shows that the cluster radius is related to the magnetic moment m_2, density ρ_p, particle radius a of a single particle and magnetic field angular velocity Ω. Comparing theoretical result and experimental data in Fig. 2E, the overall trend is followed with frequency increases while the cluster size decreases correspondingly, which confirm that the theoretical model is valid. However, theoretical and experimental values have a significant deviation in low-frequency.

Model-2 When cluster rotates vertically, considering the influence of gravity (a component of gravity along the central line $\boldsymbol{G^R}$), equation (15) can be modified as:

$$\boldsymbol{F_m^R} + \boldsymbol{G^R} = m_p\Omega^2\boldsymbol{r_{12}} \tag{19}$$

After simplification, we can get the modified cluster size.

$$R = \sqrt[3]{-\frac{s}{2} + \sqrt{\left(\frac{s}{2}\right)^2 + \left(\frac{r}{3}\right)^3}} + \sqrt[3]{-\frac{s}{2} - \sqrt{\left(\frac{s}{2}\right)^2 + \left(\frac{r}{3}\right)^3}} + \frac{gK_g}{3\Omega^2} \tag{20}$$

$$r = -\frac{g^2K_g^2}{3\Omega^4}, s = -\frac{9\mu_0 m_2^2}{8\pi^2 a^5 \rho_p \Omega^2} - \frac{2g^3K_g^3}{27\Omega^6} \tag{21}$$

Where $K_g = \cos(\theta)$ is a component of gravity along the direction of the centerline, and θ is the angle between gravity and the direction of the centerline. Therefore, R is a function with θ. Takes the average value of R during $\theta = 0\sim2\pi$, and a specific cluster size can be obtained. Moreover, comparing these two models shown in Fig. S11, model-2 shows that the change rate of cluster size at low frequency is larger than model-1 after considering gravity; in other words, gravity has a more significant impact on cluster size than the magnetic force at low frequency.

When the cluster moves horizontally near the wall, fluid resistance at the edge inhibits the cluster's size. However, since fluid resistance is related to the overall translational velocity of the cluster, the model involved is too complex, so the influence of fluid resistance on the size of the cluster is not considered in this work.

Note S3. Flow field distribution model for microfluidic flow channels with a square cross-section

Considering the low-Re fluid environment in the experimental conditions, we combine the Navier-Stokes equations with the continuity equation for viscous incompressible peristaltic flow as follows:

$$\nabla^2\overrightarrow{U_f} = \frac{1}{\mu}\left(\nabla P - \rho\vec{f}\right), \tag{22}$$

where $\vec{f}$ is the body force per unit mass. We assume that a constant pressure gradient exists within the fluid, that is:

$$\nabla P = \frac{\partial P}{\partial x} = -\frac{P_0 - P_L}{L} = \frac{-\Delta P}{L}. \tag{23}$$

The hydraulic conductivity[30] $\tilde{g}$ is:

$$\tilde{g} = \left(1 + \frac{1}{\varepsilon}\right)^2 \left[\frac{1}{3} - \frac{64}{\varepsilon\pi^5}\sum_{n=0}^{\infty}\frac{\tanh[(2n+1)\pi\varepsilon/2]}{(2n+1)^5}\right] G, \tag{24}$$

where $G = \frac{A}{L^2}$ (ratio of area to the perimeter) represents the morphological factor, and ε denotes the ratio of the side lengths for a square section $G = \frac{1}{16}$, $\varepsilon = 1$.

We define $\hat{I} = \frac{\Delta P}{L} + \rho f$. Corresponding to the volumetric flow input Q used in this study:

$$Q = \langle U_f \rangle A = \tilde{g}(\nabla P - \rho f) = -\tilde{g}\hat{I}. \tag{25}$$

Thus, deriving the fluid velocity $\langle U_f \rangle$:

$$\langle U_f \rangle = \frac{A\hat{I}}{\mu}\left[\frac{1}{12} - \frac{16}{\pi^5}\sum_{n=0}^{\infty}\frac{tanh[(2n+1)\pi/2]}{(2n+1)^5}\right], \tag{26}$$

that is:

$$U_f(x,y) = \frac{\hat{I}}{2\mu}\left[\alpha^2 - y^2 + \frac{32\alpha^2}{\pi^3}\sum_{n=0}^{\infty}\frac{(-1)^{n+1}cosh^2[(2n+1)\pi x/2\alpha]}{(2n+1)^3\cosh[(2n+1)\pi/2]}\right]. \tag{27}$$

where α denotes the side length of the flow channel section simplify this to:

$$U_f(x,y) = \frac{Q}{0.07\mu}\left[\alpha^2 - y^2 + \sum_{n=0}^{\infty}\frac{(-1)^{1+n}\cosh(\pi x^2)}{2\alpha^2(1+2n)^2}\right]. \quad (28)$$

The graph of $U_f(x,y)$ was plotted using MATLAB (Mathworks), shown in Fig. S21.

Note S4. Dynamics model of simplified VPNS upstream rolling in the Poiseuille flow

Rigid disc model The VPNS converts rotational motion into translational motion near the wall, owing to the slip-free boundary of the hydrodynamics. This means that the fluid may slide on the particle surface but not on the plain wall. This hinders the rolling of the VPNS near a surface with mismatched fluid pressure at its top and bottom, thereby inducing different velocities at each side. The snapshot from the experimental side view demonstrates that there is a highly narrow lubrication distance h between the VPNS and the wall when it is rotating upstream, which originates from the pressure difference generated by the high-speed rotation of the VPNS in the viscous fluid that makes the integral being held up by the lubricating film. Thus, it is suitable to apply the lubrication theory to analyze the forces on the VPNS. We simplify the VPNS as a microdisk of radius R and thickness t. Because motion in a super-viscous environment is overdamped, we assume a total force balance on the rotating VPNS in the Stokes state; the propulsive and drag forces in the translational direction are balanced. Moreover, the gravitational, lift, and buoyancy forces perpendicular to the propulsive direction are also balanced. Specifically, the propulsive force F_f generated by wet friction can be expressed as:

$$F_f = \mu_F F_N, \quad (29)$$

where $\mu_F = f(M,S)/R$ is the boundary friction coefficient, which is inversely proportional to the radius of the rotating disc, and $f(M,S)$ is a function of the material and surface of the target object.

The viscous resistance F_D of the microdisk model is[32], [56]:

$$F_D = 6\pi\mu R(U_p - U_f)f_1(h,R) = 2\pi\mu(U_p - U_f)t\sqrt{2R/h}, \quad (30)$$

where U_p indicates the translational velocity of the disk, and $f_1(h,R)$ is the wall correction factor related to the lubrication distance h and rotor radius R.

The forces of gravity G and buoyancy F_{By} of the VPNS are as follows:

$$G = \pi R^2 t\rho_P g \quad (31)$$

$$F_{By} = \pi R^2 t\rho_f g. \quad (32)$$

A VPNS rotating at a high angular velocity is subjected to lifts from two sources: shear lift F_{SL}[56], [26], owing to the velocity gradient of the Poiseuille flow and Saffman lift F_{RL} from autogenous rotation:

$$F_{SL} = 84.63\rho_f vt(U_p - U_f)(1/lnRes)^2 \tag{33}$$

$$F_{RL} = 2\pi\rho_f R^2 t\Omega \times (U_p - U_f), \tag{34}$$

where $Res = kR^2/v$ is the shear Reynolds number that characterizes the fluid inertia, k is the fluid shear rate, and Ω is the rotational angular velocity. Then, the dynamic equilibrium equation is established according to the force analysis:

$$\begin{cases} F_f = F_D \\ F_{SL} + F_N + F_{By} = F_{RL} + G, \end{cases} \tag{35}$$

or:

$$\begin{cases} \mu_F F_N = 2\pi\mu(U_p - U_f)t\sqrt{2R/h} \\ 84.63\rho_f vt(U_p - U_f)(1/lnRe)^2 + F_N + \pi R^2 t\rho_f g = 2\pi\rho_f R^2 t\Omega \times (U_p - U_f) + \pi R^2 t\rho_P g \end{cases}. \tag{36}$$

In the vertical direction, clusters experience Saffman lift force caused by cluster rotation, support force provided by liquid, buoyancy force comes from liquid, Magnus lift force arises from shear flow and cluster gravity. Therefore, we deduce the upstream velocity of the VPNS U_p:

$$U_p = \frac{\pi Rtf(M,S)(\rho_P - \rho_f)g}{84.63\rho_f vt(1/lnRe)^2 + 2\pi\mu t\sqrt{2R/h} - 2\pi\mu_F\rho_f R^2 t\Omega} + U_f. \tag{37}$$

The curve of the upstream migration velocity is plotted by taking the diameter of VPNS as the independent variable, as shown in Fig. S25. The gradient of the translational velocity decreases and tends towards a constant value as the swarm diameter increases. This may be owing to the faster fluid velocity at the edge of larger diameter swarms, which hinders the increase in migration velocity.

The gap between cluster and boundary As we assume above, the cluster is regarded as a homogeneous magnetic disk, whose thickness is t, the radius is R, and total magnetic moment is m_t. According to the distribution of particles in the cluster, the magnetic moment of each layer is m_2R^2/a^2, m_2 is the magnetic moment of each particle, and the number of cluster layers is $t/2R$, so the total magnetic moment is

$$m_t = Nm_2 = \frac{\pi R^2}{\pi a^2}\frac{t}{2a}m_2 = \frac{R^2 t}{2a^3}m_2 \tag{38}$$

Fig. S12A shows the forces and moments on the cluster. We can get a moment equation when balancing magnetic torque friction torque with viscous shear torque[21].

$$\frac{R^2 t}{2a^3} m_2 B - \mu_F F_N R - \frac{\mu\left(\Omega R + U_p + U_{f(2R+h)}\right)}{2R+h} AR + \frac{\mu\left(-\Omega R - U_p + U_{fh}\right)}{h} AR = 0 \tag{39}$$

Where $U_{f(2R+h)}$, $U_{f(h)}$ represents fluid velocity at the upper and lower surfaces of the cluster, respectively. For example, the velocity distribution of a one-dimensional circular tube is

$$u_f = U_f \left(1 - \left(\frac{2r}{D_f}\right)^2\right) \tag{40}$$

Where r is the distance between the cluster center and the center axis of the pipe, by solving equations (36) and (39), we can obtain the higher-order equation about h. Mathematical software can only obtain an approximate numerical solution since there is no analytic solution for a higher-order equation.

For example, $U_f = 370\ \mu\text{m/s}$, $D = 300\ \mu\text{m}$, and frequency $f = 20\sim200$ Hz are set for one-dimensional circular pipe flow. Moreover, the height between the lower surface of the cluster and the wall is shown in Fig. S12B. With the increase of frequency, the height increases gradually from 0.1352 μm to 0.2946 μm, and the corresponding cluster velocity increases from -12.65 μm/s to 119.03 μm/s.

The inset shows the cluster velocity when different height values are set. When the height increases, the same diameter's velocity also increases, indicating that height cannot be ignored while calculating the cluster velocity.

Note S5. Manipulation of clusters

According to equation (16), the cluster velocity can be derived. As we know from the above, since h is a numerical solution, so is U_p. The analytical expression of cluster velocity is listed here to show cluster velocity factors more intuitively.

We assume that the vessel center velocity is $U_f = 2$ m/s and the vessel diameter is $D_f = 2$ cm, so its velocity distribution is shown in Fig. S13A. As the frequency increases, the cluster diameter decreases rapidly to a flat level, and the cluster velocity also increases rapidly to a flat level. When the frequency reaches 2500 Hz, the cluster velocity is still negative, indicating that the cluster cannot move against the flow and can only flow with the liquid at a slow speed (Fig. S13B).

Considering the critical frequency $f_c = m_2 B/\gamma \approx 2448$ Hz of a single particle, the critical frequency of cluster synchronous movement with the magnetic field is smaller than that of a single particle. The premise of equation (19) is that the cluster rotation is synchronized with the

magnetic field; in other words, the magnetic field frequency is equivalent to the cluster frequency. When the increasing magnetic field frequency exceeds the critical cluster frequency, this equation is no longer accurate, which means the result obtained by increasing the magnetic field frequency to improve the cluster velocity is invalid.

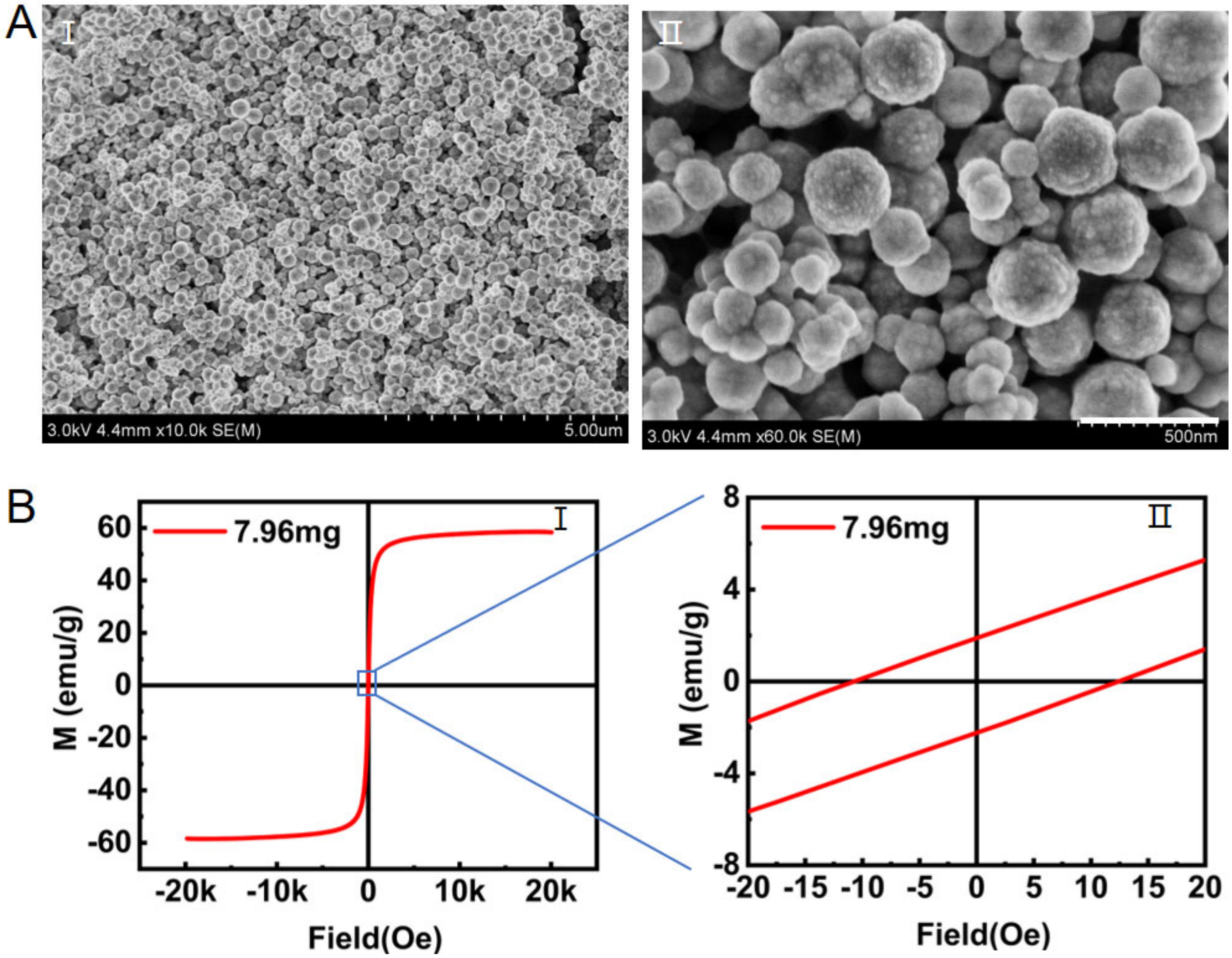

Fig. S1. Electron micrographs and magnetic characterization of MNPs. (A) Scanning electron microscope image of MNPs: (I) MNPs with an average diameter of 300 nm, magnification: 10,000×, scale bar: 5 μm. (II) Magnification: 60,000×, scale bar: 500 nm. **(B)** Paramagnetic testing of MNPs: (I) Magnetic moment variation curve of MNPs when the applied magnetic field is from -20KOe to 20KOe. (II) Hysteresis curve at a magnetic field magnitude of 20Oe.

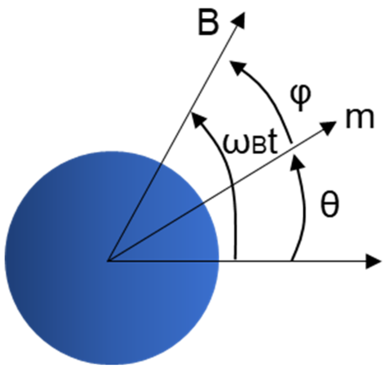

Fig. S2. Force on a single magnetic particle in a magnetic field. Phase lag (φ) between magnetic moment (m) for a spheroidal particle and magnetic field (B), ω_B is the angular velocity of the magnetic field, θ is the rotational angle of the particle.

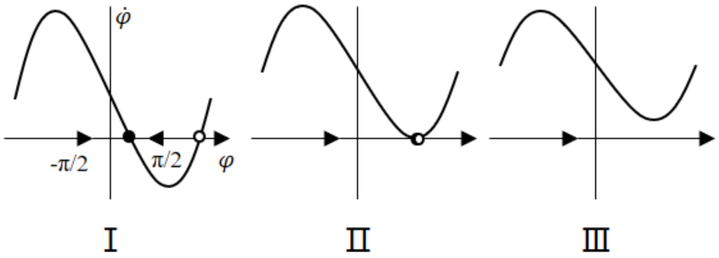

Fig. S3. Vibration state of the nonuniform oscillators system. (I) $\omega_B/\omega_c < 1$ a stable fixed point (solid) and an unstable fixed point (hollow) appear in the system. With $t \to \infty$, all trajectories are attracted to the stable fixed point. (II) $\omega_B/\omega_c = 1$ the system stops completely, and a semi-stable fixed point $\varphi = arcsin\ (\omega_B/\omega_c)$ appears at π/2. (III) $\omega_B/\omega_c > 1$ the system is unstable and showing periodic changes where fastest at -π/2 and lowest at π/2.

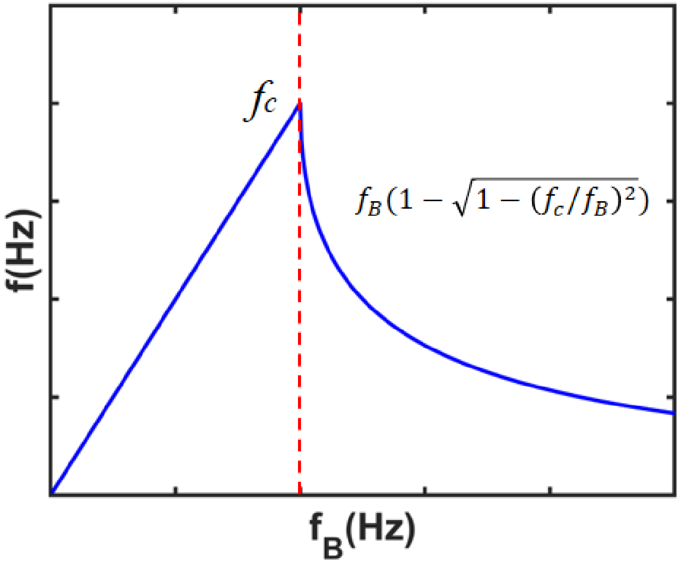

Fig. S4. Particle rotation frequency (f) and magnetic field frequency (f_B). When $f_B < f_c$, $f = f_B$. f_c is the critical frequency of a particle. When $f_B > f_c$, f decays rapidly.

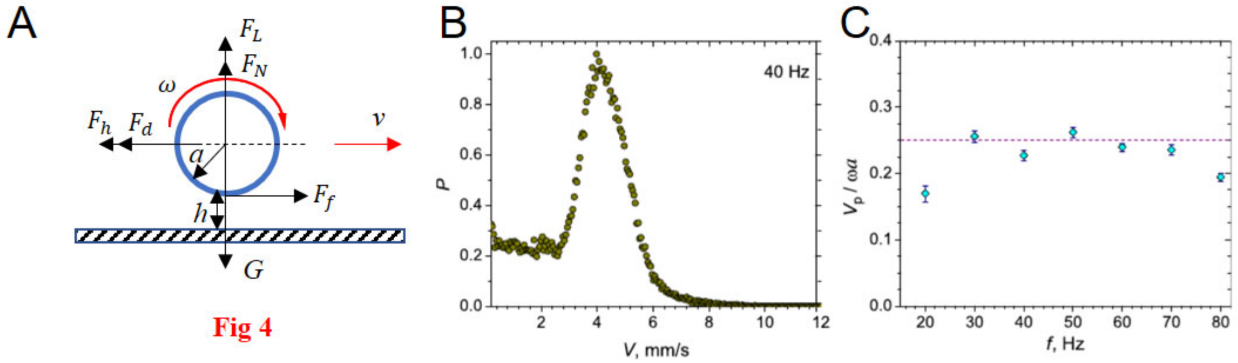

Fig. S5. Analysis of magnetic particles near the boundary. (A) Schematic diagram of particle motion near the boundary. In the vertical direction, lift force (F_L) and support force (F_N) are balanced with gravity (G). In the horizontal direction, drag force (F_h, F_d) is equal to friction. **(B)** the distribution function of particle translational velocity. **(C)** particle translational velocity and frequency. Reprinted figures with permission from ref.[53], Copyright (2017) by Science Advance.

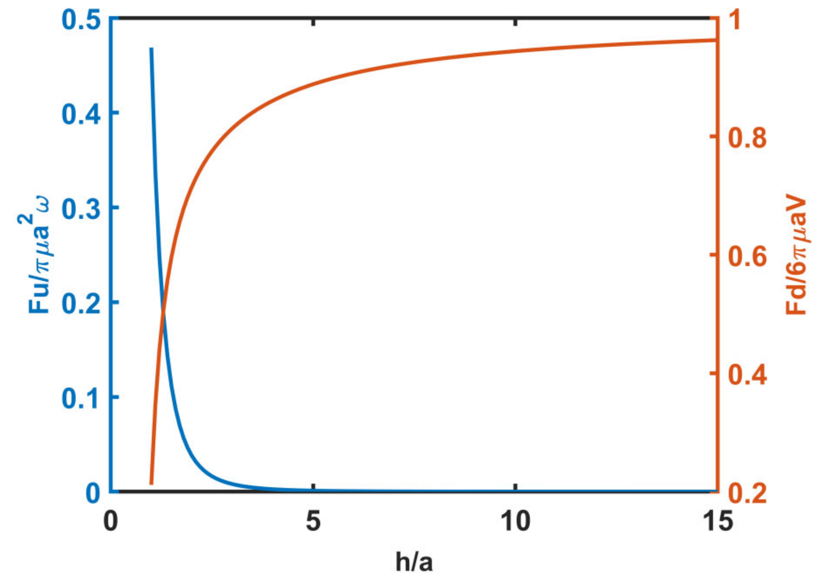

Fig. S6. Viscous force F_u and fluid drag force F_d vary with distance from the bottom surface. As h increases, normalized F_u Decreases, which means that the horizontal force due to rolling decreases. Normalized F_d becomes larger and tends to 1, which is close to the fluid resistance of a spherical particle in the unbound fluid.

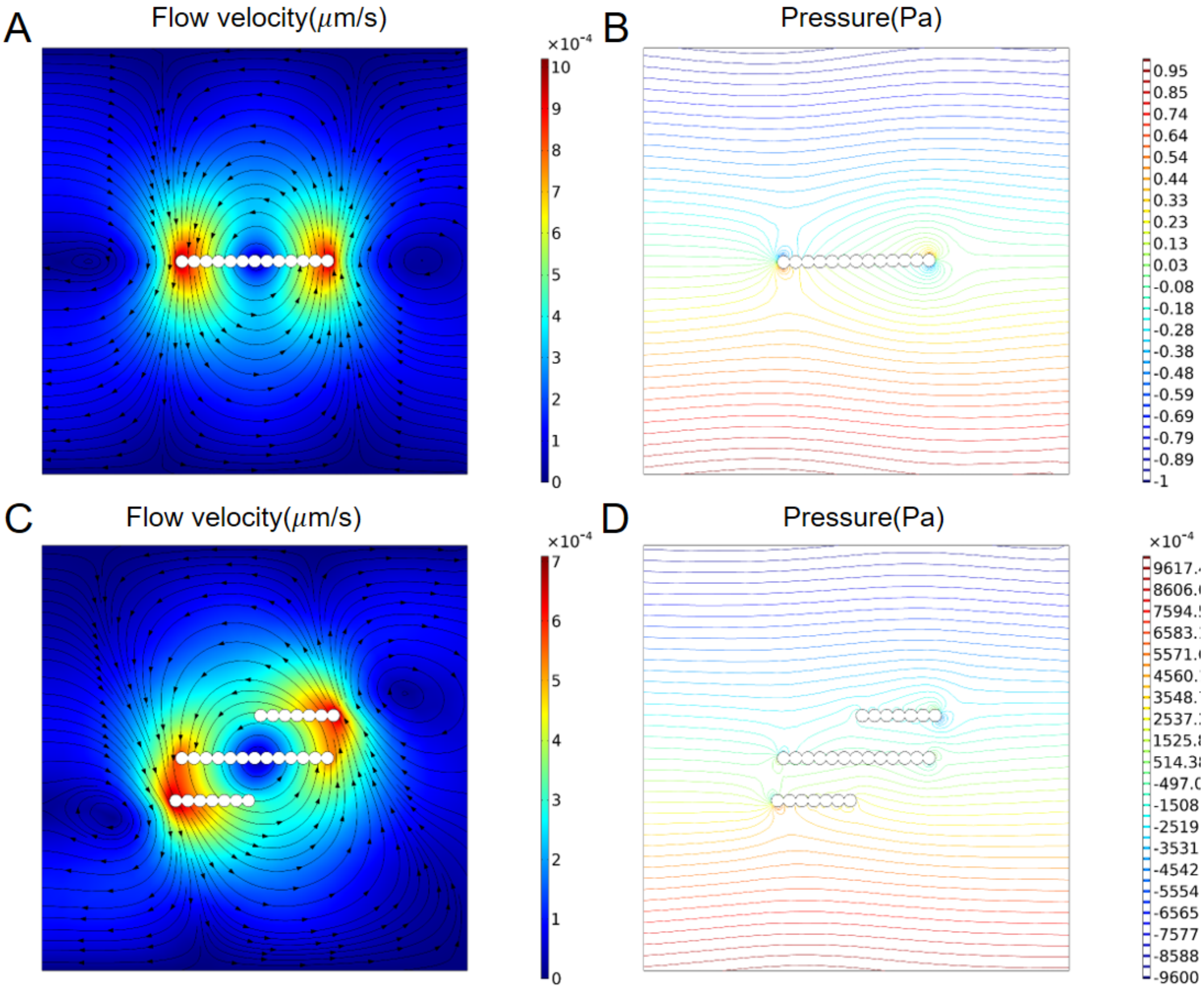

Fig. S7 Simulation of local vortices generated by short-chain rotation. (A) Flow field velocity distribution from a single rotating particle-chain-induced local vortex. **(B)** Pressure field distribution corresponding to Fig. A. **(C)** Flow field velocity distribution from multiple rotating particle-chain-induced integral vortex. **(D)** Pressure field distribution corresponding to Fig. C.

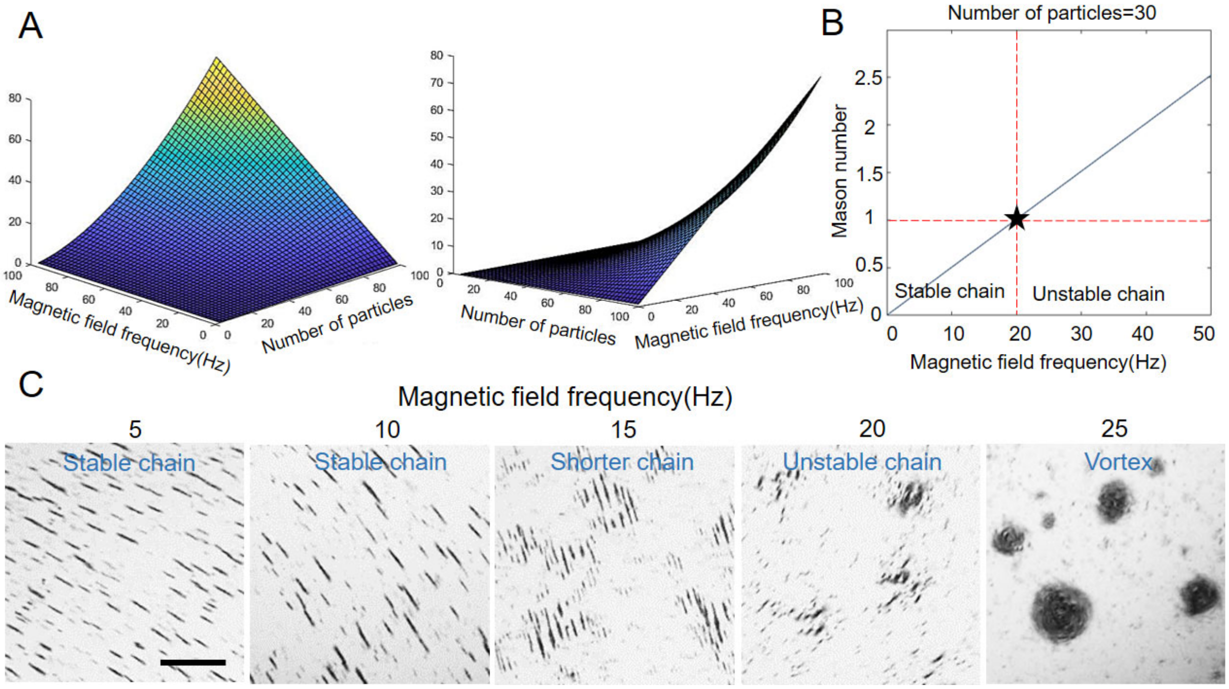

Fig. S8. Analysis of the particle chain stability. (A) Mason number versus magnetic field frequency and the number of magnetic particles. **(B)** Analytic relationship between magnetic field rotation frequency and Mason number; the critical frequency is approx. 20 Hz. **(C)** Stability states of particle chains corresponding to magnetic field frequencies under experimental conditions; the magnetic field pitch angle is 5°. The scale bar is 20 μm.

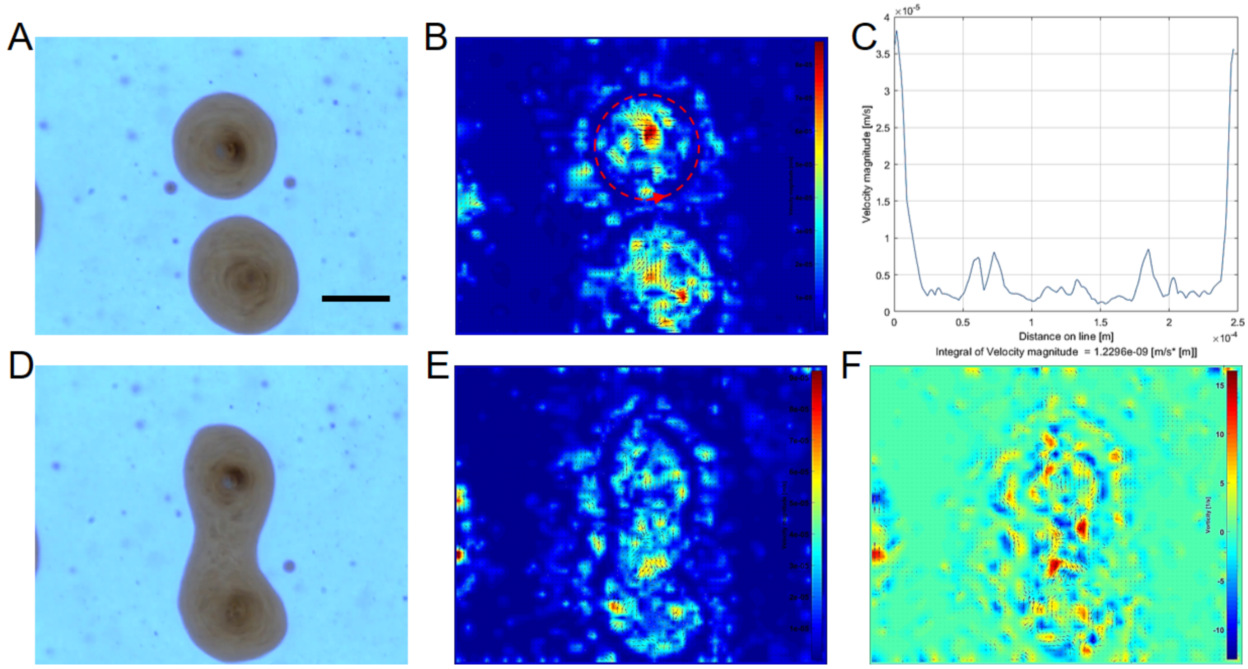

Fig. S9. Fluid analysis during the VPNS merger. (A) Two VPNSs close to each other. The scale bar is 50 μm. **(B)** Flow field analysis of two VPNSs close to each other using PIV. **(C)** The velocity of the flow field at the red dashed line in Fig. S9B. **(D)** Two merging VPNSs. **(E)** Flow field analysis of two merging VPNSs using PIV. **(F)** Vortex analysis of two merging VPNS.

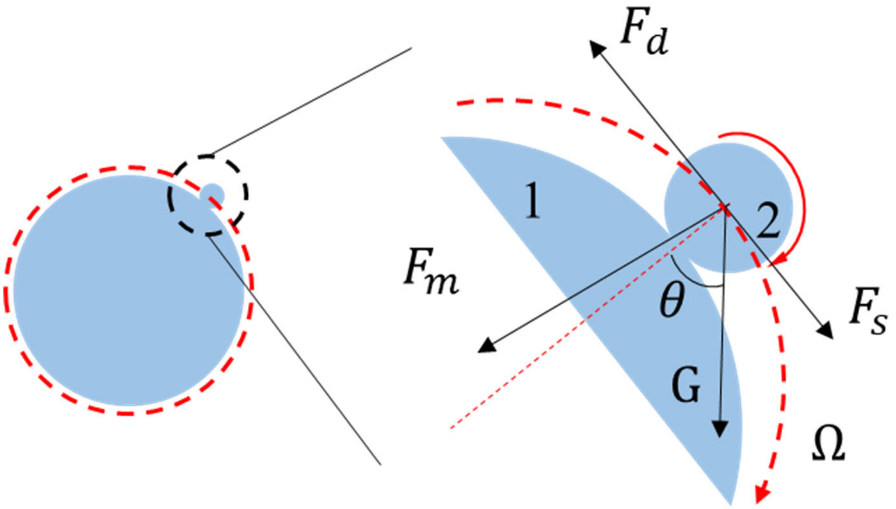

Fig. S10. Forces on the particle at the edge. F_m is the magnetic force between two magnetized particles, G is the gravity of particle 2, F_d, F_s are viscous force and shear force, respectively. Components of F_m Moreover, G along the centerline provides the centripetal force.

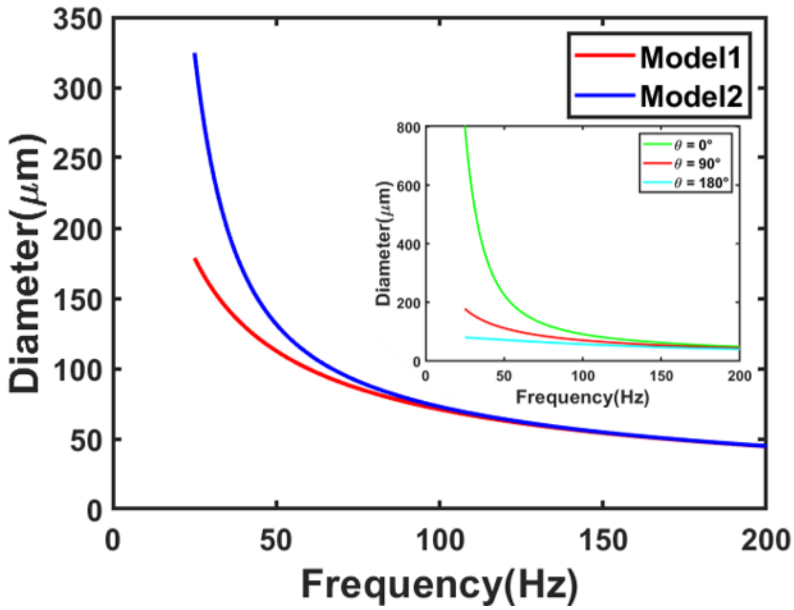

Fig. S11. Comparing of two models. Model-1 and model-2 matched well in high frequency, and model-2 is about 80% higher than model-1 in low frequency. Inset shows the cluster size (R) at different θ. As θ increases, R decreases. The blue curve is the average of all cluster sizes.

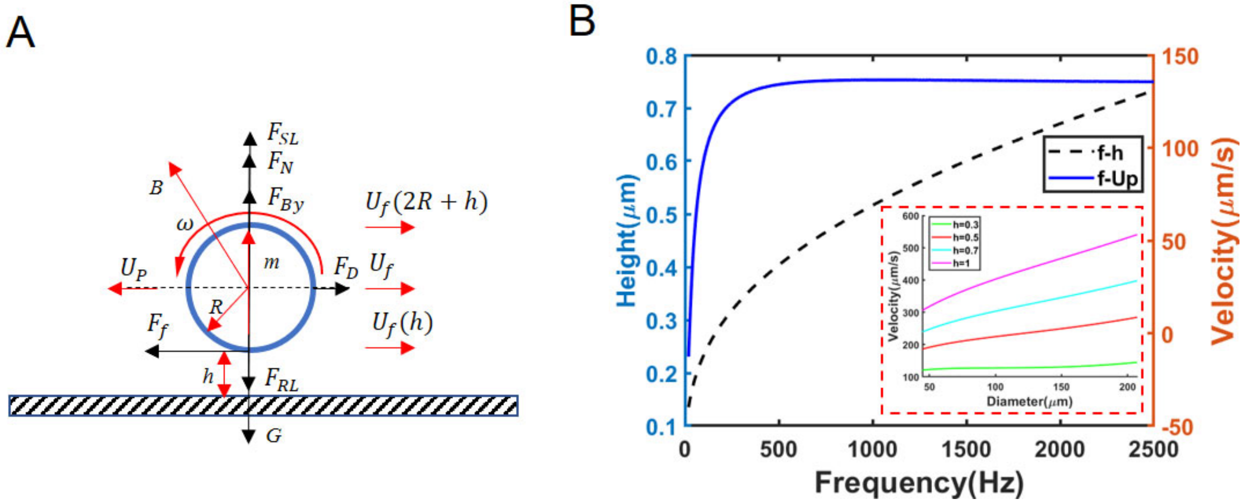

Fig. S12. Force analysis of VPNS in a dynamic flow field. (A) Forces and moments on the cluster. **(B)** Height (h) and cluster translational velocity (U_p) varies with frequency. h and U_p increase with increasing frequency. Inset shows that cluster velocity in different heights. As h increases, U_p increases for the same diameter.

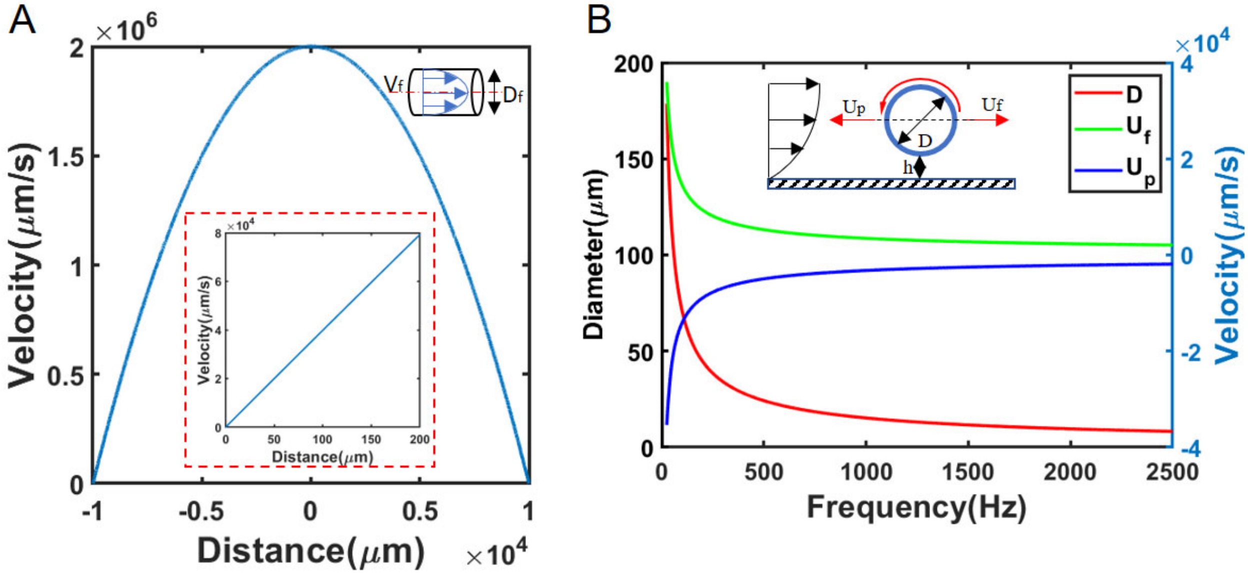

Fig. S13. Analysis of flow field velocity and cluster velocity versus frequency. (A) Distribution of flow velocity in a tube with a diameter D_f = 2 cm, center velocity U_f = 2 m/s. **(B)** cluster velocity varies with frequency. When the frequency increases from 20 Hz to 2500 Hz, cluster translational velocity is less than 0.

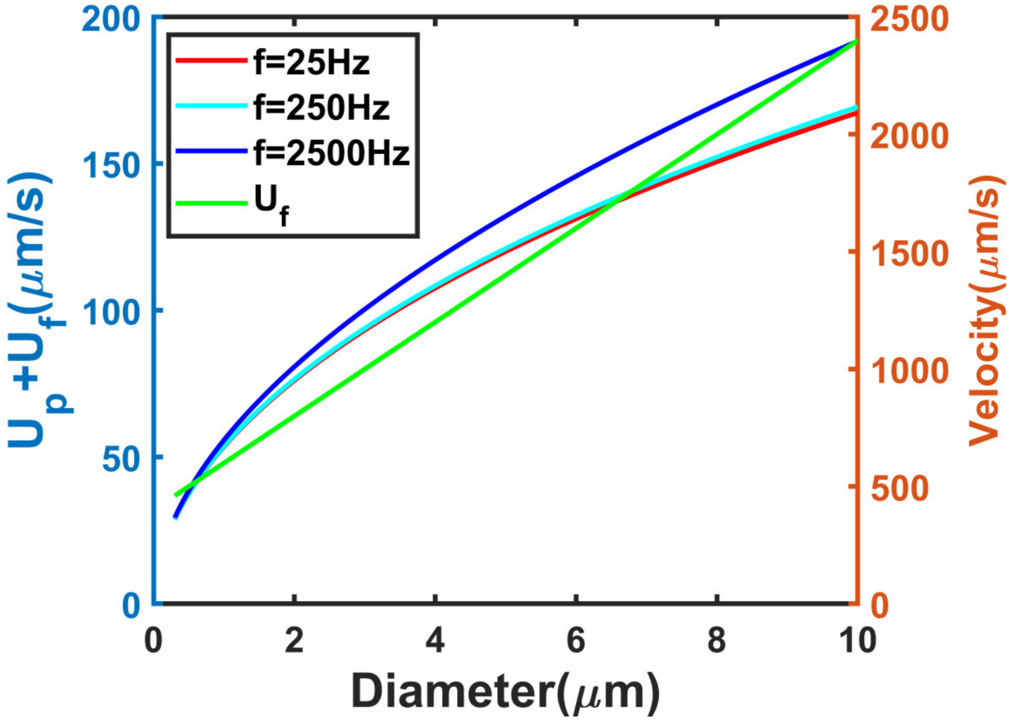

Fig. S14. Translation velocity of a single particle. With the increase of cluster size, translation velocity is only 1/10 of the fluid velocity (U_f). With the frequency increase, translation velocity is still far less than the fluid velocity.

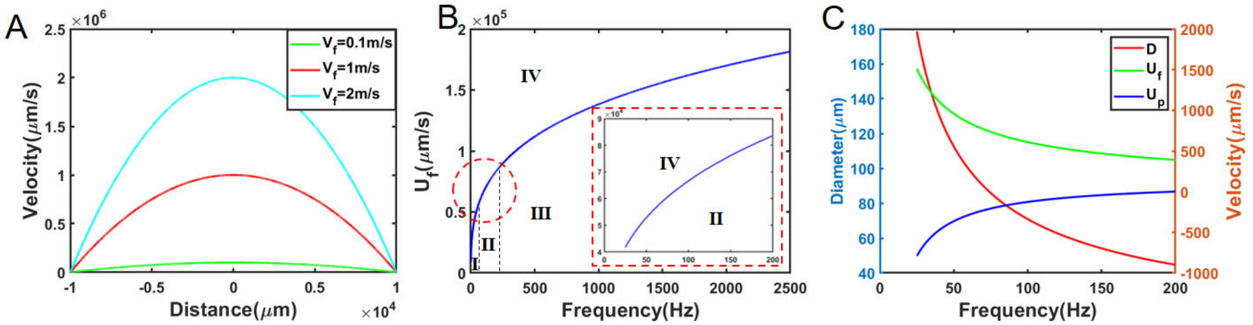

Fig. S15. Analysis of the upstream motility of VPNS in high-speed blood flow. (A) Distribution of flow velocity in the tube while central flow velocity is 0.1 m/s, 1 m/s, and 2 m/s. **(B)** Region I, particles are not easy to form clusters in lower frequency; Region II, effective region (inset f = 25~200 Hz) to let particles swim upstream; Region III, increasing relaxation of clusters leads to clusters rotation asynchronous with the magnetic field; Region IV, clusters cannot swim upstream. **(C)** Translation velocity with critical central velocity is 0.0836 m/s when the diameter is 2 cm.

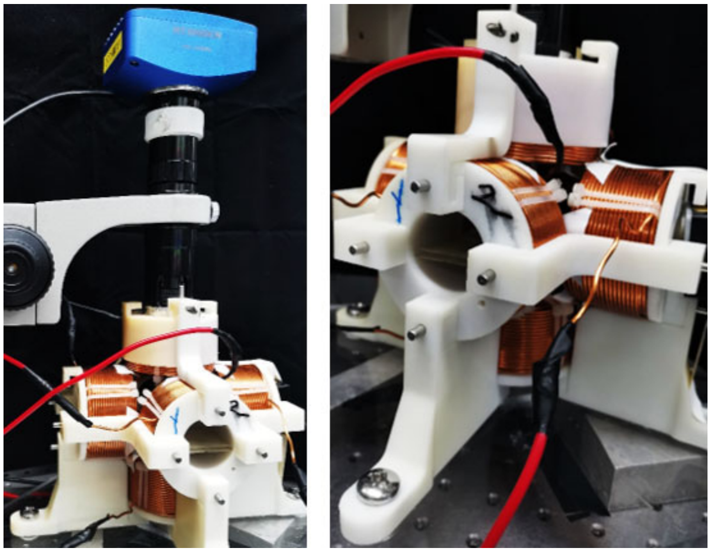

Fig. S16. The main body of the magnetic manipulation platform.

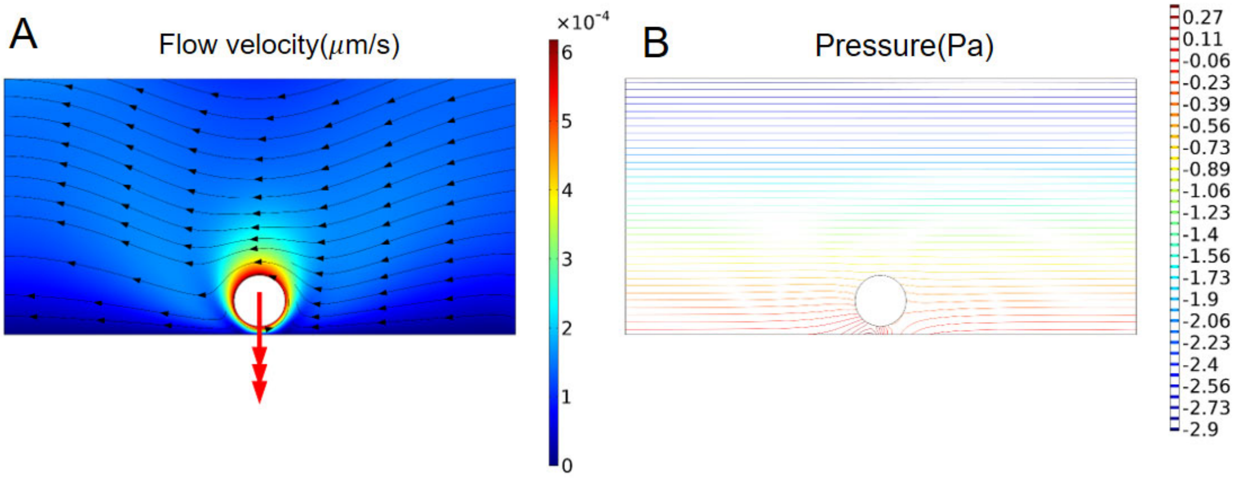

Fig. S17 VPNS wall-mounted rotational simulation. (A) Flow field distribution induced by the VPNS rotation in a liquid environment. The red arrow indicates the overall force direction obtained by integrating the circumference of the VPNS, which indicates the downward combined force on the rotating object. **(B)** Pressure field distribution corresponding to Fig. A.

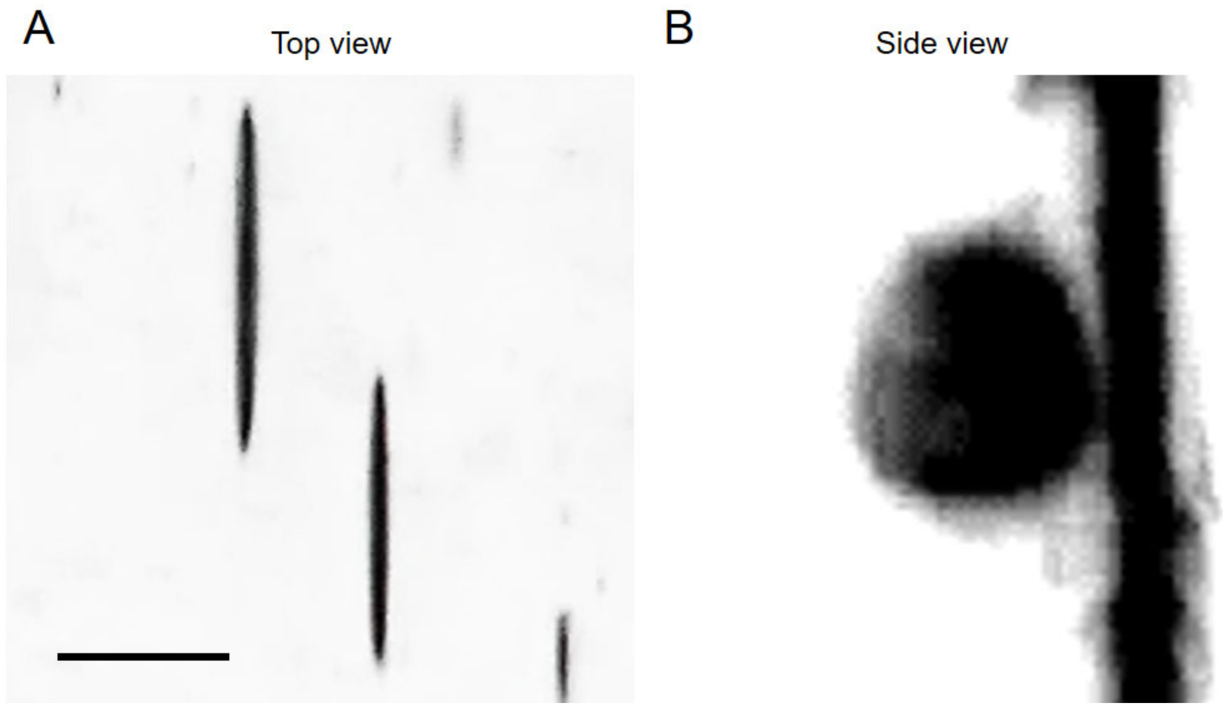

Fig. S18. Snapshot of the upstream rolling VPNS. (A) The VPNS from the top view with a very narrow shuttle shape. Scale bar is 30 μm. **(B)** The VPNS from the side view exhibits a deformable circle.

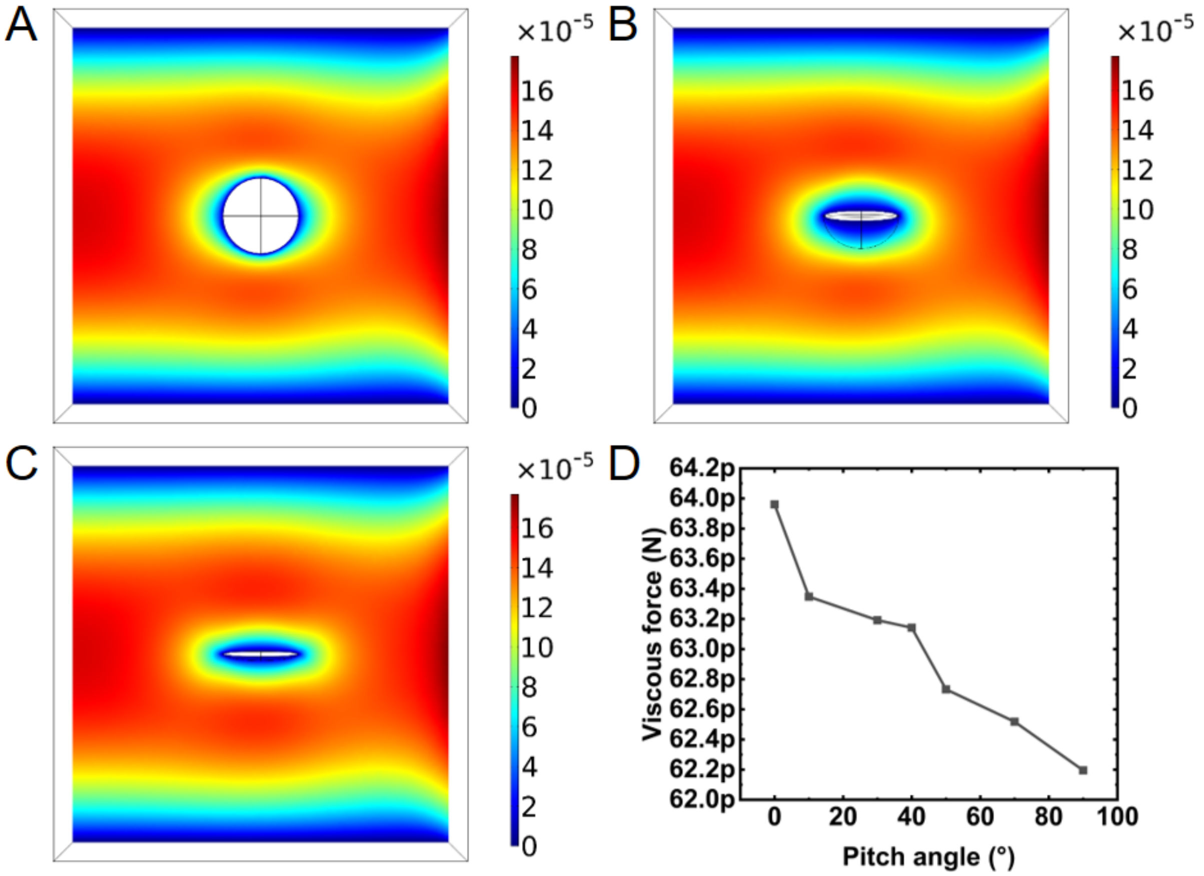

Fig. S19. Simulation analysis of VPNS viscous drag versus rolling pitch angle. (A) Pitch angle of 0°, **(B)** 30°, **(C)** 60. **(D)** Pitch angle versus viscous drag obtained by surface integration of the simulated disc.

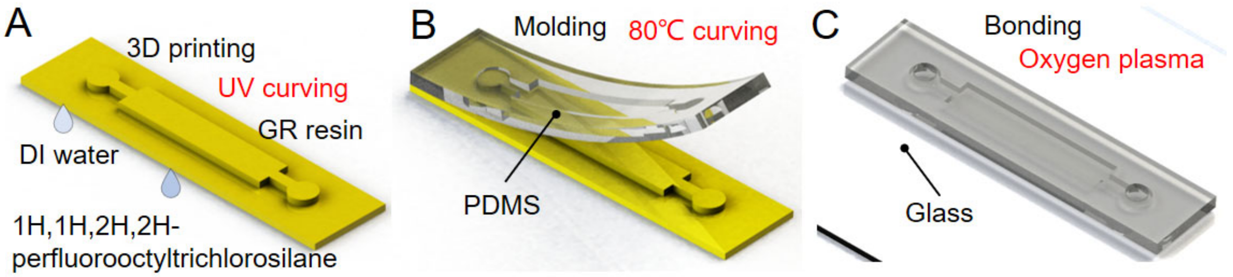

Fig. S20 Fabrication of the microfluidic channel. (A) 3D printing technology is used to cure photosensitive resin into the desired template, and a hydrophobic treatment is applied to the surface. **(B)** Casting of bubble-free PDMS onto the mold and curing at 80 °C. **(C)** Surface covalent bonding of the cured PDMS to the clean surface of the glass slides.

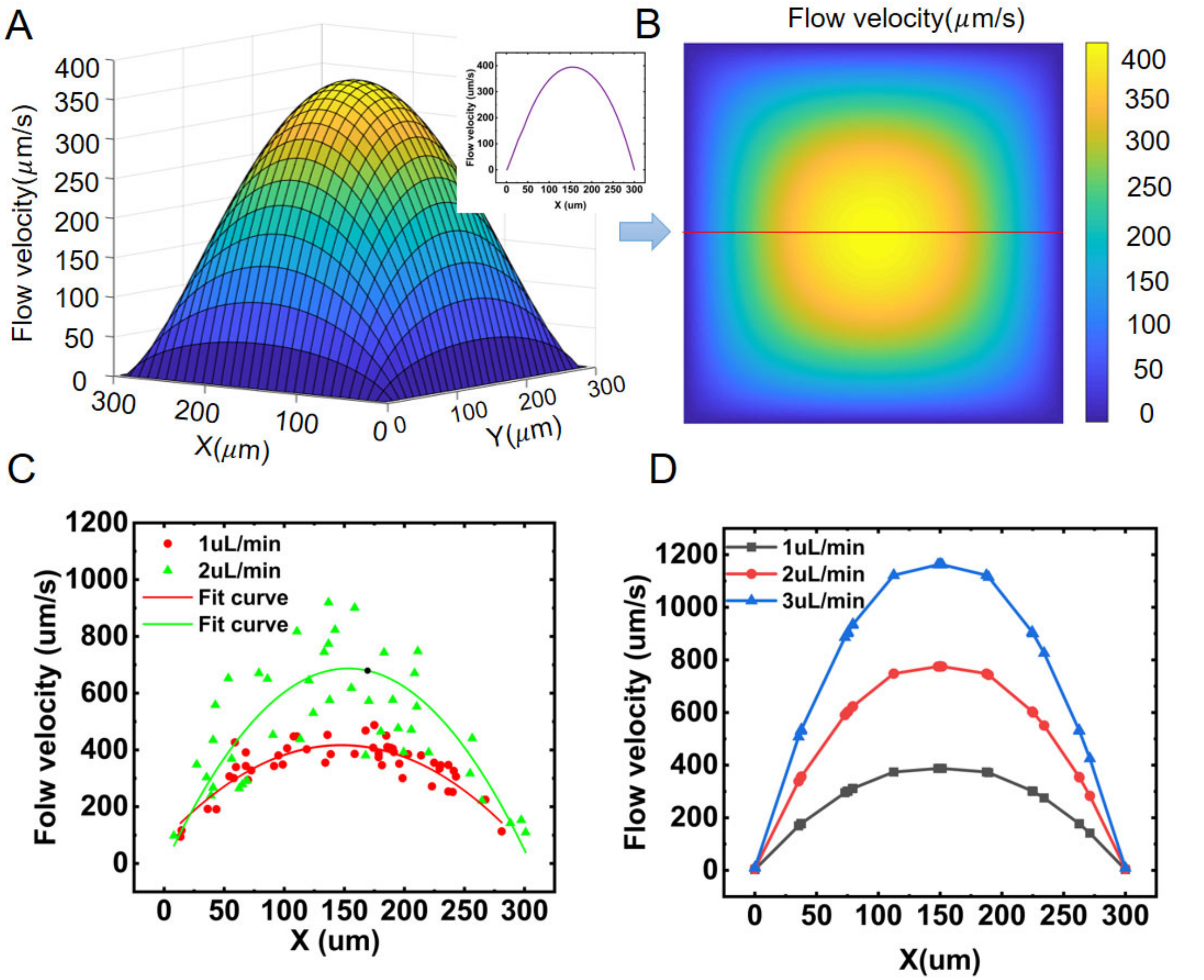

Fig. S21. Flow rate profile at higher volume flow rates. (A) Analytical surface plot of the established flow velocity distribution using MATLAB. **(B)** Flow velocity distribution in 2D sections. The inserted figure indicates the flow velocity distribution curve on a cut-off line at the center of the flow channel. **(C)** The measured flow velocities and their fitted curves at 1 μL/min and 2 μL/min volume flow rates are pumped, corresponding to the red and green parts of the graph, respectively. **(D)** The numerical solution of the flow velocities for input the volume flow rates of 1 μL/min, 2 μL/min, and 3 μL/min using COMSOL Multiphysics 5.5.

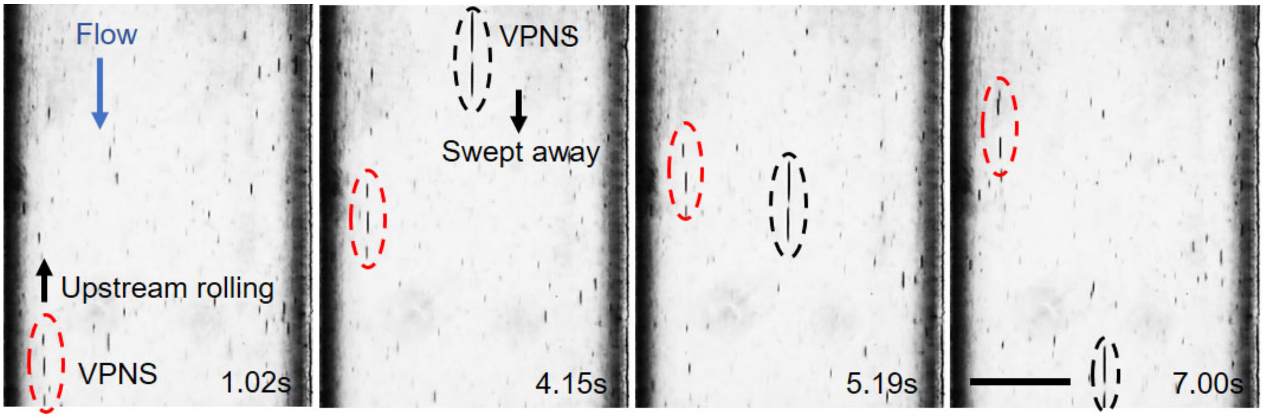

Fig. S22. Upstream motility of VPNS at higher flow rates. A smaller diameter VPNS, circled in red, can still roll upstream at the edges of the flow channel, whereas the slightly larger diameter VPNS at the center of the channel will be swept away by the high-speed fluid, as shown in the part of the figure circled in black. The scale bar is 100 μm.

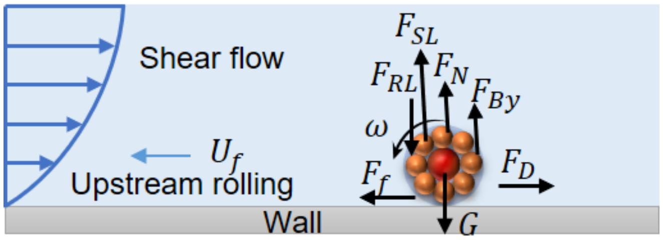

Fig. S23 Schematic diagram of the force analysis of VPNS moving upstream in the Poiseuille flow.

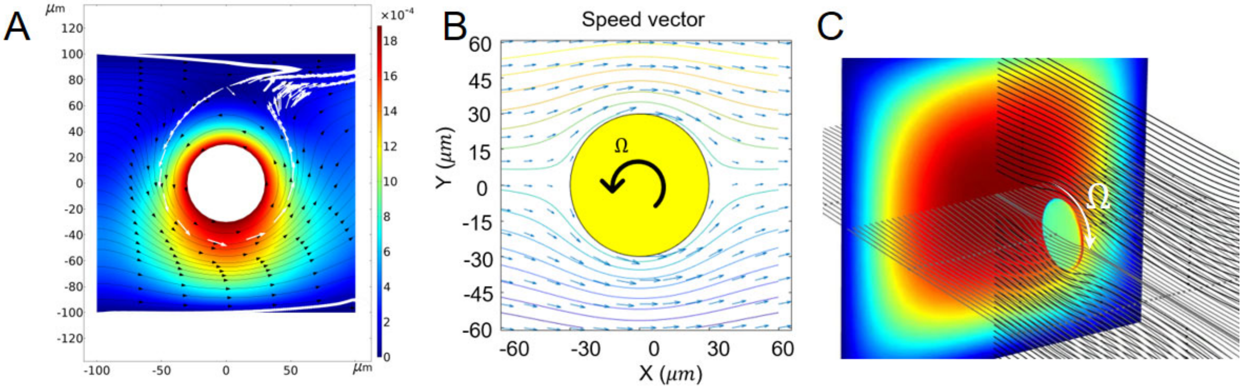

Fig. S24 Numerical and analytical analysis of the Magnus effect. (A) Simplified VPNS rotational analysis in a Poiseuille flow field using COMSOL Multiphysics 5.5. **(B)** Analysis of the simplified VPNS rotational motion using MATLAB shows the consistency of the numerical and

analytical solutions. **(C)** 3D Magnus effect simulation of the emulated microdisk under the experimental flow field, where the black line is the flow stream, and the color-gradient cloud map is the flow field distribution.

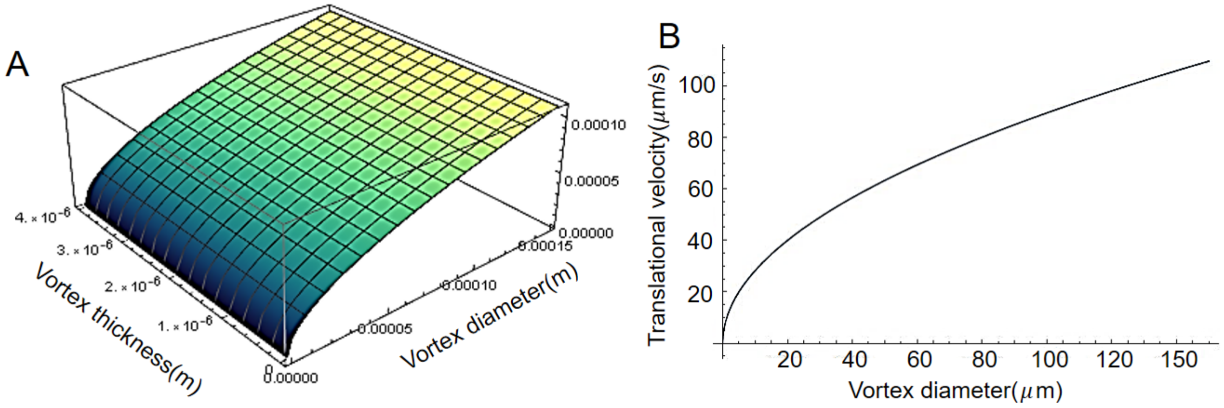

Fig. S25. Analytical relationship between the VPNS upstream velocity and diameter. (A) Plotted surfaces of VPNS upstream velocity versus its diameter and thickness. The maximum thickness of the VPNS reaches 4 μm, and the image shows that the thickness has almost no effect on the upstream velocity. **(B)** The powerful injection affects the correspondence between upstream velocity and the diameter of VPNS. The increase in vortex diameter induced the moderated increase in upstream velocity.

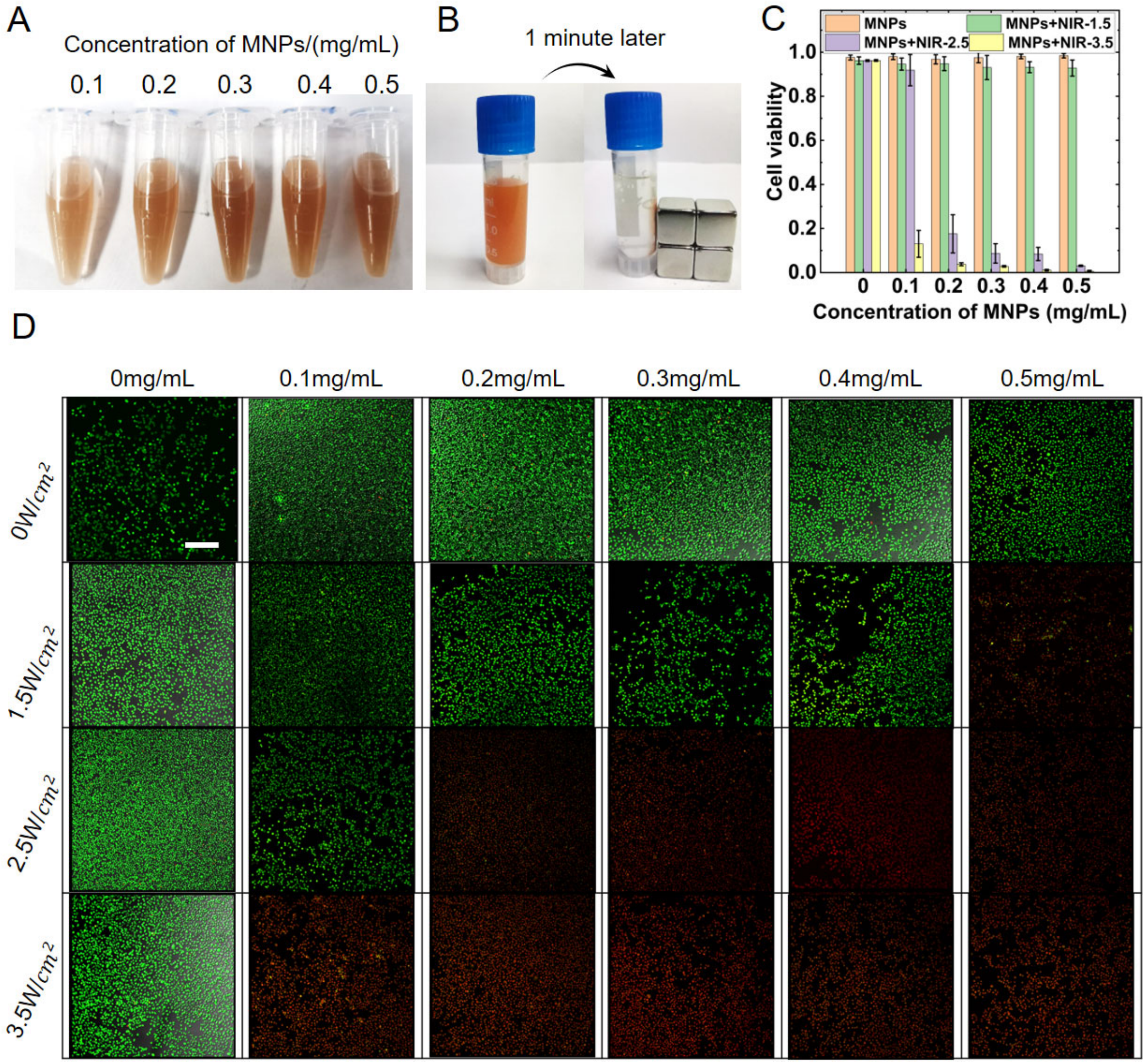

Fig. S26 The photothermal effect of MNPs induces apoptosis in HeLa cells under 1064 nm NIR irradiation for 3 min. (A) Photograph of MNP suspension at a graded mass concentration. **(B)** MNP suspension before and after 1 min of attraction with magnets. **(C)** Histogram of HeLa cell apoptosis induced by the PTT of the graded concentration of MNP suspension. **(D)** Laser scanning confocal micrographs of HeLa cells co-stained with calcein-AM and PI, in which live cells are stained with green fluorescence by calcein-AM, and dead cells are stained with red fluorescence by PI. The scale bar is 200 μm.

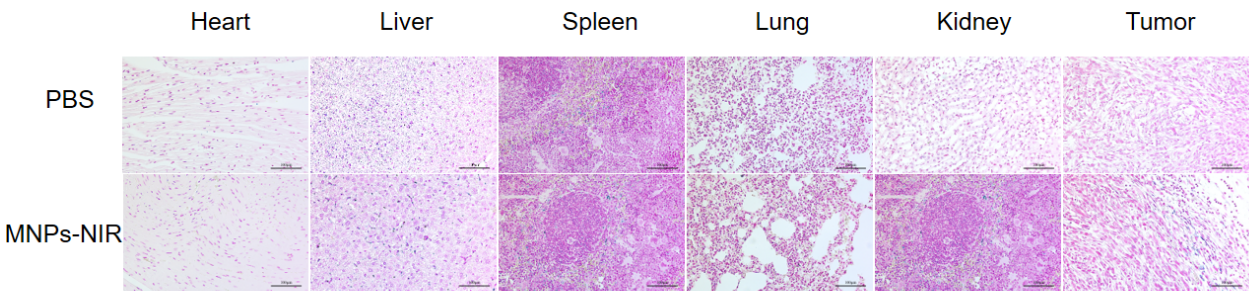

Fig. S27 Prussian blue staining images of major organs in PBS and MNPs-NIR groups.

Table S1. Parameters used in the theoretical analysis

Parameter	Value
Radius of MNPs-a	0.3×10^{-6} [m]
Radius of VPNS-R	28×10^{-6} [m]
Microfluidic channel side length-α	150×10^{-6} [m]
Thick of VPNS-t	4×10^{-6} [m]
Nanoparticle density-ρ_p	5180 [kg/m^3]
water density-ρ_f	1000 [kg/m^3]
Gravity acceleration-g	9.8 [m/s^2]
Water dynamic viscosity-μ	1.01×10^{-3} [Pa·s]
Water kinematic viscosity-ν	1.01×10^{-6} [m^2/s]
Microfluidic chip side length-L	300×10^{-6} [m]
Boltzmann constant-K_B	1.380649×10^{-23} [J/K]
Absolute temperature-T	298.15 [K]
Fluid average velocity-U_f	370×10^{-6} [m/s]
Vortex swarm average velocity-U_p measured	100×10^{-6} [m/s]
Clearance height-h	1×10^{-6} [m/s]
Permeability in a vacuum-μ_0	$4\pi \times 10^{-7}$ [$N \cdot A^{-2}$]
Magnetic field strength-H	6×10^{-3} [T]
Maximum magnetization rate$-\chi$ measured	58.54058 [emu/g]
Reynolds number-Re	0.1099
